# Transcription and Translation Products of the Cytolysin Gene *psm-mec* on the Mobile Genetic Element SCC*mec* Regulate *Staphylococcus aureus* Virulence

**DOI:** 10.1371/journal.ppat.1001267

**Published:** 2011-02-03

**Authors:** Chikara Kaito, Yuki Saito, Gentaro Nagano, Mariko Ikuo, Yosuke Omae, Yuichi Hanada, Xiao Han, Kyoko Kuwahara-Arai, Tomomi Hishinuma, Tadashi Baba, Teruyo Ito, Keiichi Hiramatsu, Kazuhisa Sekimizu

**Affiliations:** 1 Laboratory of Microbiology, Graduate School of Pharmaceutical Sciences, The University of Tokyo, Tokyo, Japan; 2 Department of Infection Control Science, Graduate School of Medicine, Juntendo University, Tokyo, Japan; Dartmouth Medical School, United States of America

## Abstract

The F region downstream of the *mecI* gene in the SCC*mec* element in hospital-associated methicillin-resistant *Staphylococcus aureus* (HA-MRSA) contains two bidirectionally overlapping open reading frames (ORFs), the *fudoh* ORF and the *psm-mec* ORF. The *psm-mec* ORF encodes a cytolysin, phenol-soluble modulin (PSM)-mec. Transformation of the F region into the Newman strain, which is a methicillin-sensitive *S. aureus* (MSSA) strain, or into the MW2 (USA400) and FRP3757 (USA300) strains, which are community-acquired MRSA (CA-MRSA) strains that lack the F region, attenuated their virulence in a mouse systemic infection model. Introducing the F region to these strains suppressed colony-spreading activity and PSMα production, and promoted biofilm formation. By producing mutations into the *psm-mec* ORF, we revealed that (i) both the transcription and translation products of the *psm-mec* ORF suppressed colony-spreading activity and promoted biofilm formation; and (ii) the transcription product of the *psm-mec* ORF, but not its translation product, decreased PSMα production. These findings suggest that both the *psm-mec* transcript, acting as a regulatory RNA, and the PSM-mec protein encoded by the gene on the mobile genetic element SCC*mec* regulate the virulence of *Staphylococcus aureus*.

## Author Summary

Methicillin-resistant *Staphylococcus aureus* (MRSA) causes opportunistic infectious diseases in immunocompromised patients in hospitals, which are difficult to cure with antibiotics. These MRSA are called hospital-associated MRSA (HA-MRSA). In contrast, other MRSA strains, which are called community-acquired MRSA (CA-MRSA), have high virulence and cause serious infections in previously healthy individuals. Why the CA-MRSA phenotype has high virulence is not well understood. MRSA becomes resistant to antibiotics by acquiring a foreign DNA called SCC*mec*. The SCC*mec* region contains several genes, including the *mecA* gene, which confers resistance against methicillin. We focused our attention on the structural difference of the SCC*mec* region between HA-MRSA and CA-MRSA. The SCC*mec* region of CA-MRSA lacks the F region, which suppresses HA-MRSA virulence. In this study, we revealed that both the transcription and translation products of a cytolysin gene, *psm-mec*, which is encoded in the F region, suppress MRSA virulence. This finding contributes to our understanding of the high virulence of CA-MRSA at the molecular level and will help to establish new strategies to combat infectious diseases due to CA-MRSA.

## Introduction

*Staphylococcus aureus* is a pathogenic bacterium that causes various diseases in humans. The emergence of methicillin resistant *S. aureus* (MRSA), vancomycin resistant *S. aureus*, and community acquired MRSA (CA-MRSA) is a serious clinical problem [1], [2], [3], [4]. These MRSA species are thought to have evolved by acquiring mobile genetic elements that carry antibiotic resistance genes or virulence genes [5]. CA-MRSA is more virulent than hospital-associated MRSA (HA-MRSA) [4], [6], which is isolated from hospitalized patients or patients having risk factors for HA-MRSA, such as catheter use, recent surgery, drug use, etc. [7], [8]. The different virulence phenotypes of these two MRSAs is suggested to be due to Panton-Valentine leukocidin (PVL) [9], [10], which is encoded on lysogenized bacteriophages, a mobile genetic element [11], or phenol-soluble modulin α (PSMα), which is encoded in the core genome [12]. The *psmα* operon exists in all *S. aureus* genomes sequenced to date and encodes PSMα1, α2, α3, and α4, with lytic activity against neutrophils [12]. The expression of *psmα* is elevated in most prevalent CA-MRSA strains, including LAC (USA300) and MW2 (USA400) [12]. The *psmα-*deleted mutant of CA-MRSA strains exhibits attenuated virulence in a mouse systemic infection model and a mouse skin infection model [12]. The molecular mechanisms that cause the elevated expression of PSMα protein in CA-MRSA strains, however, are not known.

We previously reported that *S. aureus* possesses the ability to spread on soft agar plates, a process we called “colony spreading” [13], which requires cell wall teichoic acids [13] and a cell envelope-associated protein, MsrR [14]. HA-MRSA strains have low colony-spreading ability, whereas CA-MRSA strains have high colony-spreading ability [15]. The structure of SCC*mec*, a mobile genetic element that confers methicillin resistance to MRSA strains, is different between HA-MRSA and CA-MRSA strains [16]. In a type-II SCC*mec* element, we identified a genomic region that explains the difference in the colony-spreading ability between CA-MRSA and HA-MRSA, locating downstream of the *mecI* ORF (+285 to +859 from the translational initiation site of *mecI*) [15]. We call this region the “F region” in the present study. The F region exists in type-II and type-III SCC*mec*, which are found on most HA-MRSA, but not in the type-IV SCC*mec*, which is found in most CA-MRSA [15]. Introduction of the F region into Newman, a methicillin-sensitive *S. aureus* (MSSA) strain, suppresses the colony spreading and attenuates virulence in a mouse systemic infection model [15]. The 575-bp F region contains an open reading frame (ORF) that putatively encodes 70 amino acids, which we named *fudoh* [15]. Recently, Queck *et al.* reported that the *psm-mec* ORF, which encodes a cytolytic peptide, exists on the opposite strand of the *fudoh* ORF [17].

In the present study, we aimed to clarify the molecular mechanism of the F region by which *S. aureus* virulence is decreased and found that the F region suppresses PSMα production and promotes biofilm formation. Thus, we hypothesized that the absence of the F region in CA-MRSA strains is a reason for the elevated PSMα protein production. Furthermore, to examine which ORFs in the F region are responsible for the *S. aureus* phenotype, we introduced stop codon mutations into the *fudoh* and *psm-mec* ORFs and found that both the transcription and translation products of the *psm-mec* ORF contribute to negatively regulate staphylococcal virulence, whereas the *fudoh* ORF does not contribute to suppress colony spreading and virulence.

## Results

### The *fudoh* ORF is not necessary to suppress colony spreading

**Figure 1:**
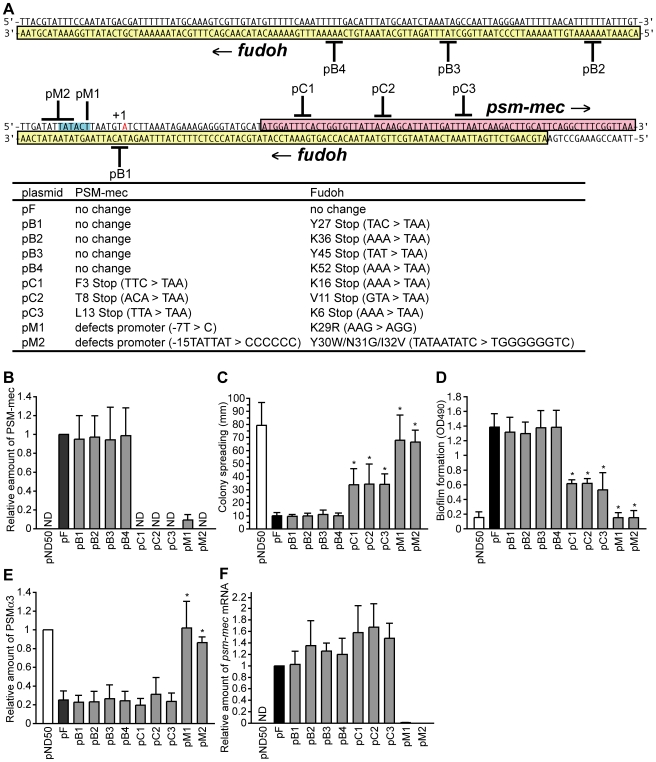
Analysis of nucleotide substitutions in the F region. (A) Nucleotide sequences of the *psm-mec* ORF and *fudoh* ORF are shown. The *psm-mec* ORF (magenta-colored) is encoded from left to right, whereas the *fudoh* ORF (yellow-colored) is encoded from right to left. Black bold lines indicate the substituted nucleotides. Red-colored nucleotide indicates the transcription start site (+1) of the *psm-mec* ORF, which was determined in this study (Fig. S3). Blue-colored nucleotides are a putative −10 region for transcription of the *psm-mec* ORF. Table shows the amino acid substitutions caused by the nucleotide substitutions. For pM1 and pM2, the nucleotide substitutions are presented, numbered from transcription start site. (B) The PSM-mec productions of Newman strains transformed with various plasmids harboring nucleotide substitutions in the *psm-mec* ORF and *fudoh* ORF were examined by HPLC. The data are presented as the means ± standard deviations from at least three independent experiments. ND, not detected. (C) The colony-spreading abilities of Newman strains transformed with various plasmids harboring nucleotide substitutions in the *psm-mec* ORF and *fudoh* ORF were examined. Plates were incubated for 8 h at 37°C and the means ± standard deviations of the halo diameters from at least three independent experiments are shown. The asterisk indicates a p-value of less than 0.05, calculated by Student's t-test, between the sample and the pF-transformed Newman strain. (D) Biofilm formations on polystyrene microplates of Newman strains transformed with various plasmids harboring nucleotide substitutions in the *psm-mec* ORF and *fudoh* ORF were examined. The asterisk indicates a p-value of less than 0.05, calculated with the Student's t-test, between the sample and the pF-transformed Newman strain. (E) The PSMα3 production of Newman strains transformed with various plasmids harboring nucleotide substitutions in the *psm-mec* ORF and *fudoh* ORF was examined by HPLC. The data shown represent the means ± standard deviations from at least three independent experiments. The asterisk indicates a p-value of less than 0.05, calculated with Student's t-test, between the sample and the pF-transformed Newman strain. (F) The amounts of the *psm-mec* mRNA in Newman strains transformed with various plasmids harboring nucleotide substitutions in the *psm-mec* ORF and *fudoh* ORF were measured by quantitative reverse transcription-PCR. The data are presented as the means ± standard deviations from at least three independent experiments. ND, not detected.

We previously proposed that the *fudoh* ORF, located downstream of the *mecI* gene in the type-II SCC*mec* region, suppresses *S. aureus* colony spreading [15]. The existence of the *psm-mec* ORF (69 bp) in the opposite strand of the *fudoh* ORF (210 bp) was recently reported (Fig. 1A) [17]. The 575-bp F region contains these two ORFs on the opposite strands. We constructed base substitution mutations of the F region to clarify whether the *fudoh* ORF or *psm-mec* ORF inhibits colony-spreading activity.

pB1, pB2, pB3, and pB4 contain stop codons in the *fudoh* ORF that do not alter the amino acid sequence of the translation product of the *psm-mec* ORF (Fig. 1A). pC1, pC2, and pC3, however, contain stop codons in both the *psm-mec* ORF and *fudoh* ORF (Fig. 1A). We measured the amount of the *psm-mec* ORF translation product in the bacterial strains transformed with these plasmids by an established method using HPLC [17]. Newman strains transformed with pB1, pB2, pB3, and pB4, which harbor the same *psm-mec* coding sequence as pF, produced amounts of PSM-mec comparable to those in the pF-transformed Newman strain (Fig. 1B). On the other hand, Newman strains transformed with pC1, pC2, and pC3 did not produce PSM-mec (Fig. 1B).

Newman strains transformed with pC1, pC2, and pC3 showed more colony spreading than the pF-transformed Newman strain, although their colony spreading was much less than that of the vector (pND50)-transformed Newman strain (Fig. 1C). On the other hand, the colony spreading of Newman strains transformed with pB1, pB2, pB3, and pB4, which contain stop codons in the *fudoh* ORF, were suppressed to levels similar to those of the pF-transformed Newman strain (Fig. 1C). Thus, interruption of translation of the *fudoh* ORF by stop codons did not affect the inhibition of colony spreading (pB1, pB2, pB3, pB4), whereas interruption of the translation of the *psm-mec* ORF by stop codons attenuated the inhibition of colony spreading (pC1, pC2, pC3). These results suggest that colony spreading is not inhibited by the translation product of the *fudoh* ORF, but rather by the PSM-mec protein, the translation product of the *psm-mec* ORF. There were also no differences between pF-transformed Newman and pB1, pB2, pB3, and pB4-transformed Newman strains in the F-dependent phenotypes that were newly discovered and are explained later in this manuscript (Fig. 1D, E). Moreover, we did not detect transcripts and translation products of the *fudoh* ORF in pF-transformed Newman strain (data not shown), indicating that the *fudoh* ORF is not functional. Interruption of the translation of the *psm-mec* ORF by stop codons (pC1, pC2, pC3) did not completely abolish the inhibition of colony spreading, indicating that factors other than the translated product of the *psm-mec* ORF contributed to inhibit colony spreading.

### Introduction of the F region into MSSA and CA-MRSA strains decreased their mouse-killing ability, colony-spreading ability, and the production of extracellular PSMs, but promoted biofilm formation

**Figure 2:**
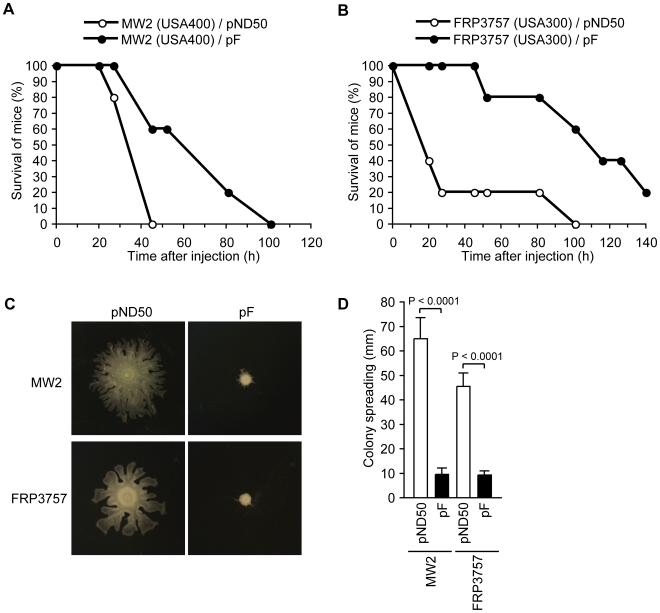
Introduction of the F region into CA-MRSA strains attenuates virulence in a mouse systemic infection model and decreases colony-spreading ability. (A) CD-1 mice (n = 5) were intravenously injected with MW2 transformed with pND50 or pF (2×10^8^ CFU) and survival was monitored. Statistical analysis was performed with the Kaplan-Meier test. The P-value between pND50 and pF is 0.0411. (B) CD-1 mice (n = 5) were intravenously injected with FRP3757 transformed with pND50 or pF (2×10^8^ CFU) and survival was monitored. Statistical analysis was performed with the Kaplan-Meier test. The P-value between pND50 and pF is 0.0142. (C) Overnight cultures of MW2 harboring pND50 or pF and FRP3757 harboring pND50 or pF were spotted onto soft agar plates and incubated for 8 h at 37°C. (D) The means ± standard deviations of the halo diameters of at least three independent experiments are presented.

To understand the effect of the F region against *S. aureus* virulence, we examined the phenotypes of *S. aureus* strains that were transformed with the F region. In a mouse systemic infection model, mice injected with the F region-introduced MW2 (USA400) strain or the F region-introduced FRP3757 (USA300) strain, which are CA-MRSA strains, survived longer than mice injected with empty vector-introduced parent strains (Fig. 2A, B). Therefore, the F region decreased the virulence of the MW2 and FRP3757 strains in mice. We then examined whether introducing the F region into CA-MRSA strains decreases colony-spreading ability. Both the F region-introduced MW2 and F region-introduced FRP3757 strains showed decreased colony-spreading ability compared with the empty vector-introduced parent strains (Fig. 2C, D). These results suggest that absence of the F region underlies the virulence of CA-MRSA strains in causing systemic diseases as well as colony spreading ability.

**Figure 3:**
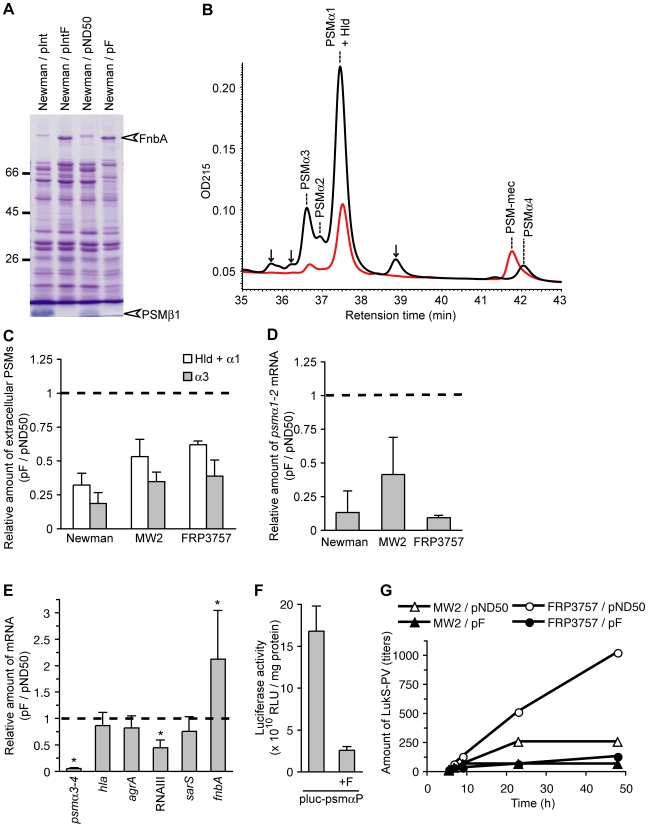
Introduction of the F region decreases the amount of extracellular PSMs and increases the amount of extracellular FnbA. (A) The Newman strain was transformed with an integration plasmid pInt, pInt harboring the F region (pIntF), a multicopy plasmid pND50, or pND50 harboring the F region (pF). Extracellular proteins at the stationary phase were separated by 12.5% sodium dodecyl sulfate-polyacrylamide gel electrophoresis and stained with Coomassie brilliant blue. The white arrowhead indicates the excised band for LC-tandem MS analysis and was identified as FnbA and PSMβ1 (Table S1). (B) Measurement of the amount of extracellular PSMs by HPLC. Overnight cultures of the Newman strain harboring pND50 (black line) or pF (red line) were subjected to HPLC and absorbance at 215 nm was obtained. Respective PSMs were identified by LC/MS (Fig. S2). Hld and PSMα1 were contained in the same peak in this assay condition. Arrows indicate unidentified molecules. (C) Amount of PSMs in the pF-transformed strain relative to that in the pND50-transformed strain in Newman, FRP3757, and MW2 genetic backgrounds is presented. (D) Expression of the *psmα1-2* mRNA was measured by quantitative reverse transcription-PCR (qRT-PCR) in Newman, FRP3757, and MW2 strains. Amount of the *psmα1-2* mRNA in the pF-transformed strains relative to that in the pND50-transformed strains is presented. (E) Expression of the *psmα3-4*, *hla*, *agrA*, RNAIII, *sarS*, and *fnbA* were measured by qRT-PCR in pF-transformed and pND50-transformed Newman strains. The asterisks indicate a p-value of less than 0.05, calculated with Student's t test, between pND50- and the pF-transformed Newman strains. (F) Promoter activity of the *psmα* operon was measured by a luciferase-based reporter assay in the Newman strain. The Newman strain was transformed with pluc-psmαP or pluc-psmαP-F. The means ± standard deviations of three independent experiments are presented. (G) Amounts of LukS-PV during growth in brain heart infusion (BHI) medium were measured. Cells were cultured in 10 ml BHI-medium using an Advantec TN2612 photorecorder. Aliquots of the culture were centrifuged at 3000 rpm for 20 min, and amounts of PVL in the culture supernatant were estimated using anti-LukS-PV monoclonal antibody-coated latex particles, developed by Denka Seiken, Co. Ltd, Niigata, Japan [51]. Representative data from three experiments are shown.

*S. aureus* produces various extracellular proteins that affect its virulence [18], [19]. To understand the molecular mechanism underlying the effect of the F region to suppress mouse systemic infection and colony spreading, we tested our hypothesis that introducing the F region affects the expression of extracellular proteins. Analysis of exoproteins of F region-introduced Newman, an MSSA strain, by sodium-dodecyl sulfate polyacrylamide gel electrophoresis revealed an increase in the amount of a 90-kDa protein, whereas the amount of a protein that migrated faster than the tracking dye was decreased compared with the empty vector-introduced Newman strain (Fig. 3A). Liquid chromatography-tandem mass spectrometry experiments identified the 90-kDa protein as fibronectin binding protein A (FnbA) and the lower molecular-weight protein as a cytolytic peptide, PSMβ1 [12] (Table S1). MSSA produces PSM subtypes α1, α2, α3, α4, β1, β2, and Hld, which are all small hydrophobic polypeptides [12]. We examined whether introducing the F region into the Newman strain affected the amount of these PSMs by high performance liquid chromatography (HPLC) analysis of the culture supernatants [12], [20]. Not only the amount of PSMβ1, but also the amounts of α1+Hld, α2, α3, and α4 were decreased in the culture supernatants of the F region-introduced Newman strain (Fig. 3B, C). To determine whether the decrease in the amount of PSMαs and the increase in FnbA was due to the altered amount of mRNA, we measured the amount of the transcript of the *psmα* operon and the *fnbA* gene in the F region-introduced Newman strain by quantitative reverse transcription-polymerase chain reaction (qRT-PCR) analysis. In the F region-introduced Newman strain, the amounts of *psmα1-2* and *psmα3-4* mRNA were decreased to much less than that in the empty vector-introduced Newman strain (Fig. 3D, E), indicating that the decrease in PSMαs induced by introducing the F region was caused by a decrease in the amount of *psmα* mRNA. The amount of *fnbA* mRNA was increased in the F region-introduced Newman strain (Fig. 3E), indicating that the increase in extracellular FnbA is caused by an increase in the amount of *fnbA* mRNA. To examine whether decreased expression of the *psmα* mRNA in the F region-introduced Newman strain was caused by decreased promoter activity, we measured the *psmα* promoter activity. The *psmα* promoter activity was decreased in the F region-introduced Newman strain (Fig. 3F), indicating that the F region decreased transcriptional initiation of the *psmα* operon. To examine whether the F-region affects the expression of other virulence genes, we also measured the amount of transcripts of the *hla* gene encoding α-hemolysin; RNAIII, which is a regulatory RNA transcribed from the *agr* locus and globally regulates virulence gene expression [21]; *agrA*, a response regulator that positively regulates *psmα* expression [22]; and *sarS*, a transcription factor for virulence genes [23], [24], [25]. Although *hla*, *agrA*, and *sarS* expression was not altered, the amount of RNAIII was decreased in the F region-introduced Newman strain compared with the empty vector-introduced Newman strain (Fig. 3E), indicating that the F-region has an inhibitory effect on the RNAIII regulation of virulence genes.

Furthermore, we examined whether introducing the F region into CA-MRSA strains that lack the F region decreased PSMα production, as in case of the Newman strain. The F region-introduced MW2 (USA400) and F region-introduced FRP3757 (USA300) strains produced less PSMαs in the culture supernatant and the amount of *psmα1-2* mRNA was less than that in empty vector-introduced parent strains (Fig. 3C, D). In addition, we examined whether the production of PVL, a cytolytic toxin composed of LukS-PV and LukF-PV that is one of the virulent determinants of the CA-MRSA strains, is influenced by the presence of the F-region. FRP3757 and MW2 strains carrying pF produced smaller amount of LukS-PV than those carrying an empty vector (Fig. 3G). Thus, the absence of the F region underlies the increased expression of PSMαs and PVL in CA-MRSA strains.

**Figure 4:**
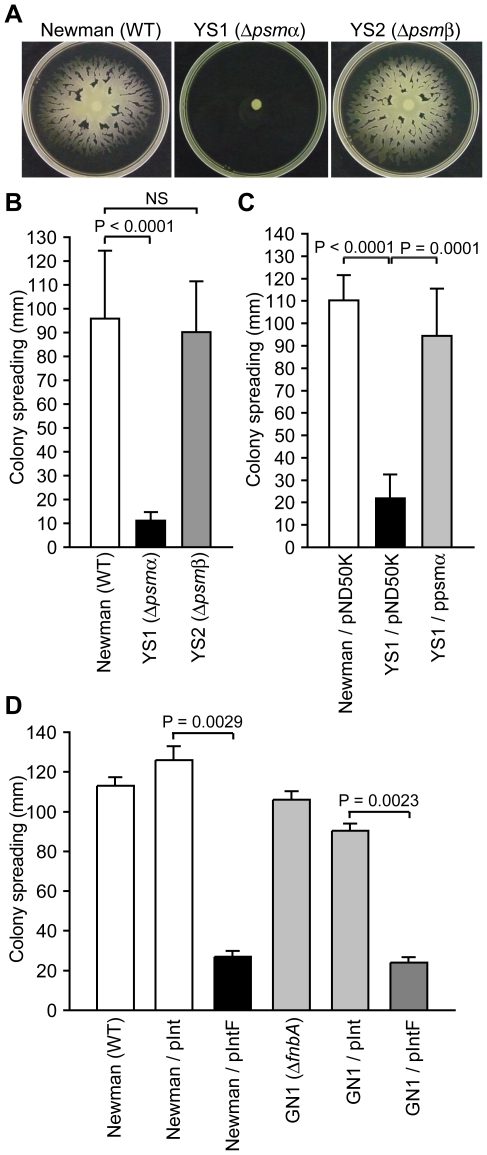
The *psmα* operon is required for colony spreading. (A) Overnight cultures of Newman, YS1 (Δ*psmα*), and YS2 (Δ*psmβ*) were spotted onto soft agar plates and incubated for 10 h at 37°C. (B) The means ± standard deviations of the halo diameters of at least three independent experiments are presented. (C) YS1 was transformed with pND50K or ppsmα. Overnight cultures were spotted onto soft agar plates and incubated for 10 h at 37°C. The halo diameters are presented. (D) The colony-spreading ability of Newman, the *fnbA*-disrupted mutant (GN1), and the F region introduced GN1 was examined. The halo diameters are presented.

To determine whether the decreased amount of extracellular PSMs in the F region-introduced Newman and CA-MRSA strains contributed to the decreased colony-spreading ability, we examined colony-spreading of null mutants for the *psmα* operon encoding PSMα1, PSMα2, PSMα3, and PSMα4, and for the *psmβ* operon encoding PSMβ1 and PSMβ2. The *psmα*-deleted mutant did not show colony-spreading activity (Fig. 4A, B). Moreover, introduction of the plasmid harboring the *psmα* operon restored colony-spreading ability of the *psmα*-deleted mutant (Fig. 4C). These results indicate that the *psmα* operon is required for *S. aureus* colony spreading. Thus, the decreased expression of the *psmα* operon is at least one reason for the decreased colony-spreading ability of the F region-introduced Newman or CA-MRSA strains. In contrast, the *psmβ*-deleted mutant showed colony-spreading activity similar to that of the parent strain (Fig. 4A, B). Thus, a decrease in the amount of PSMβ1 did not contribute to decrease the colony-spreading ability.

Next, to determine whether the increase in the amount of extracellular FnbA in the F region-introduced Newman strain decreased colony-spreading ability, we constructed an *fnbA-*disrupted mutant. The *fnbA-*disrupted mutant exhibited colony-spreading activity similar to that of the parent strain (Fig. 4D). In addition, introduction of the F region into the *fnbA-*disrupted mutant decreased the colony-spreading ability to a level similar to that in the parent strain (Fig. 4D). Therefore, the increased amount of extracellular FnbA caused by introduction of the F region did not contribute to decrease colony-spreading ability.

**Figure 5:**
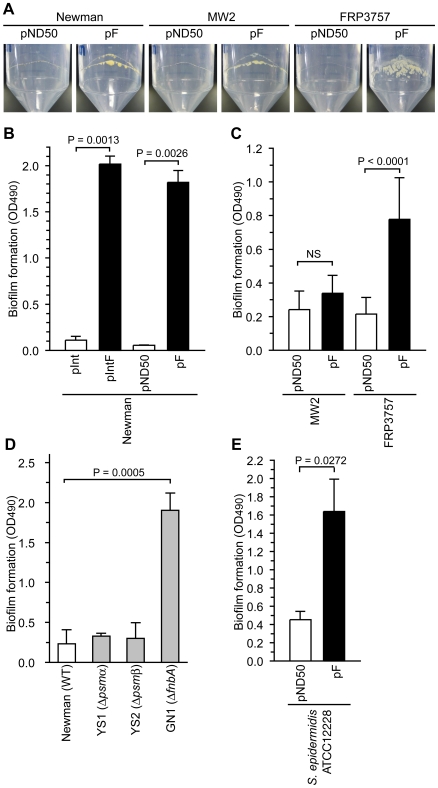
Introduction of the F region promotes biofilm formation. (A) Newman harboring pND50 or pF, MW2 harboring pND50 or pF, and FRP3757 harboring pND50 or pF were cultured in a 50-ml polypropylene tube for 3 days. After removing bacterial cultures, the bacterial adherence to the inner surface of the tubes was observed. (B) The Newman strain was transformed with an integration plasmid pInt, pInt harboring the F region (pIntF), a multicopy plasmid pND50, or pND50 harboring the F region (pF). The bacterial strains were cultured in polystyrene microplates and the bacterial cells that adhered to the plates were stained with safranin. The OD_490_ was measured. (C) Biofilm formation of MW2 harboring pND50 or pF and FRP3757 harboring pND50 and pF onto polystyrene microplates was examined. (D) Biofilm formation of Newman strain, the *psmα*-deleted mutant (YS1), the *psmβ*-deleted mutant (YS2), and the *fnbA*-disrupted mutant (GN1) onto polystyrene microplates was measured. (E) Biofilm formation of *S. epidermidis* ATCC12228 harboring pND50 or pF onto polystyrene microplates was measured.

Our previous observation that water in soft agar plates stimulates colony spreading of *S. aureus* and that a gene responsible for synthesizing cell wall teichoic acids is required for colony-spreading suggests that the interaction between the cell surface and the soft agar surface is important for colony spreading [13]. Our findings that introduction of the F region into Newman and CA-MRSA strains suppressed colony-spreading and altered the expression of extracellular proteins suggest that the presence of the F region affects the extracellular environment and cell surface structure. The extracellular environment and cell surface structure affects biofilm formation, which is an important phenotype for bacterial pathogenicity [26], [27], [28]. *S. aureus* forms a biofilm on polypropylene medical devices [29]. Introduction of the F region into the Newman, MW2, and FRP3757 strains promoted bacterial adherence on the internal surfaces of polypropylene tubes (Fig. 5A). We also examined the effect of the F region on *S. aureus* biofilm formation using polystyrene microplates. Introduction of the F region into the Newman and FRP3757 strains promoted biofilm formation on polystyrene, whereas the F region-introduced MW2 strain did not show increased biofilm formation (Fig. 5B, C). Thus, the F region-promoted *S. aureus* biofilm formation on polystyrene is dependent on the genetic background.

To determine whether the decrease in the amount of PSMαs and PSMβ1, or the increase in the amount of FnbA in the F region-introduced Newman strain promotes biofilm formation, we examined biofilm formation of *psmα*, *psmβ*, and *fnbA* mutants. Both the *psmα*-deletion mutant and the *psmβ*-deletion mutant formed low levels of biofilm that were indistinguishable from that of the parent strain (Fig. 5D). Therefore, the increased biofilm formation by the F region-introduced Newman strain was not due to the decrease in PSMαs and PSMβ1. On the other hand, the *fnbA*-disrupted mutant had higher biofilm formation than the parent strain (Fig. 5D). Thus, the *fnbA* gene repressed biofilm formation in the Newman strain. This means that the increased biofilm formation in the F region-introduced Newman strain was not due to the increased amount of extracellular FnbA. These results suggest that biofilm formation promoted by the F region was caused by mechanisms other than the expression of *psmα*, *psmβ*, and *fnbA*. The Newman strain has a truncated *fnbA* gene and secretes FnbA, which is a rare phenotype among *S. aureus* strains [30]. The negative effect of *fnbA* on biofilm formation might be due to truncation of the *fnbA* gene in the Newman strain.

### PSM-mec, a translation product of the *psm-mec* ORF encoded in the F region, contributes to promote biofilm formation, but not to inhibit PSMα production

**Figure 6:**
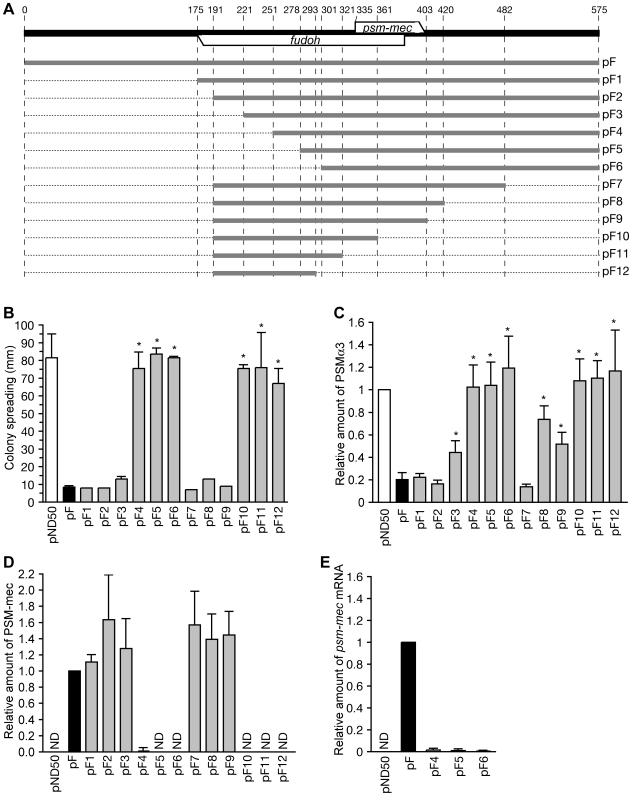
Analysis of domain deletions of the F region. (A) The 575-bp F region is indicated by a bold black line. The *fudoh* ORF exists at the opposite strand of the *psm-mec* ORF. Domain deletions of the F region indicated by bold grey lines were cloned into plasmids. The names of the plasmids are shown on the right side. (B) The colony-spreading abilities of Newman strains transformed with various plasmids harboring domain deletions of the F region were examined. Plates were incubated for 8 h at 37°C and the means ± standard deviations of the halo diameters from at least three independent experiments are shown. The asterisk indicates a p-value of less than 0.05, calculated with Student's t-test, between the sample and the pF-transformed Newman strain. (C) The PSMα3 productions of Newman strains transformed with various plasmids harboring domain deletions of the F region were examined by HPLC. The data were the means ± standard deviations from at least three independent experiments. The asterisks indicate a p-value of less than 0.05, calculated with Student's t test, between the sample and the pF-transformed Newman strain. (D) PSM-mec production of the Newman strains transformed with various plasmids harboring domain deletions of the F region were examined by HPLC. The data were the means ± standard deviations from at least three independent experiments. ND, not detected. (E) The amounts of the *psm-mec* mRNA in Newman strains transformed with pF4, pF5, and pF6 were measured by qRT-PCR. The data are presented as the means ± standard deviations from at least three independent experiments.

We constructed various types of domain deletions of the F region (Fig. 6A) and base substitution mutations of the F region (Fig. 1A) to clarify whether the translation product of *psm-mec* ORF inhibits PSMα production, and stimulates the biofilm formation caused by introduction of the F region.

pF3 with deletion of 0–221 bp and pF9 with deletion of 403–575 bp inhibited colony spreading as well as pF harboring the intact F region (Fig. 6B). In contrast, pF4 with deletion of 0–251 bp and pF10 with deletion of 361–575 bp did not inhibit colony-spreading activity (Fig. 6B). Therefore, the 221–251 bp and 361–403 bp regions are required to inhibit colony spreading. Although pF3 with deletion of 0–221 bp and pF8 with deletion of 420–575 bp inhibited colony-spreading activity to the same extent as pF (Fig. 6B), these plasmids decreased the inhibition of PSMα production (α3, Fig. 6C; Hld + α1, Fig. S1A). In contrast, pF2 with deletion of 0–191 bp and pF7 with deletion of 482–575 bp inhibited PSMα production (α3, Fig. 6C; Hld + α1, Fig. S1A). Therefore, neither the 191–221 bp region nor the 420–482 bp region, which locate outside of the *psm-mec* ORF, were required to inhibit colony spreading, but contributed to inhibit PSMα production.

Although Newman strains transformed with pF1, pF2, pF3, pF7, pF8, and pF9 produced the same amount of PSM-mec as the Newman strain transformed with pF, Newman strains transformed with pF4, pF5, pF6, pF10, pF11, and pF12 produced little PSM-mec (Fig. 6D). The Newman strains transformed with pF4, pF5, and pF6 (Fig. 6E) contained little *psm-mec* mRNA, indicating that the 221–251 bp-region (84–114 nt upstream of the translation start of the *psm-mec*) is important for the transcription of *psm-mec*. Thus, the colony-spreading inhibition correlated with the amount of PSM-mec. This finding confirms that the PSM-mec protein was involved in the inhibition of colony spreading, as indicated by base substitution experiments (Fig. 1C). In contrast, the inhibition of PSMα production did not correlate with the amount of PSM-mec in strains transformed with pF3, pF8, and pF9, indicating that factors other than PSM-mec inhibited PSMα production.

We also examined stop codon mutations into the *psm-mec* ORF of the F region (Fig. 1A, pC1; pC2; pC3) and attempted to determine the factor in the F region responsible for inhibiting PSMα production, and for stimulating biofilm formation. Newman strains transformed with pC1, pC2, and pC3 showed decreased PSMα production, similar to pF-transformed Newman (α3, Fig. 1E; Hld+α1, Fig. S1B). This result suggests that the factor that inhibits PSMα production is not the translation product of the *psm-mec* ORF.

Newman strains transformed with pC1, pC2, and pC3 formed less biofilm than the pF-transformed Newman, although much more biofilm was formed than in the empty vector-transformed Newman strain (Fig. 1D). This result suggests that the translation product of the *psm-mec* ORF, PSM-mec, promoted biofilm formation and that factors other than the translation product of the *psm-mec* ORF also contributed to stimulate biofilm formation.

### Transcription product of the *psm-mec* ORF inhibits PSMα production as a regulatory RNA molecule

We previously demonstrated that pM1 harboring the base displacement of −33T with C from the translation start of the *psm-mec* ORF lost inhibition of colony spreading [15] (Fig. 1A and 1C). The mutated nucleotide locates outside of the *psm-mec* ORF. We examined the possibility that the nucleotide substitution affects the expression of the *psm-mec* ORF. The pM1-transformed Newman strain produced little PSM-mec compared with the pF-transformed Newman strain (Fig. 1B). Moreover, the amount of *psm-mec* mRNA was considerably lower in the pM1-transformed Newman compared with the pF-transformed Newman strain (Fig. 1F). Therefore, the loss of the colony spreading inhibitory activity in pM1 was due to the inhibition of transcription of the *psm-mec* ORF by the mutation. The pM1-transformed Newman strain showed completely restored colony-spreading ability, in contrast to the Newman strain transformed with pC1, pC2, or pC3 (Fig. 1C). This finding led us to hypothesize that not only the translation product but also the transcription product of the *psm-mec* ORF contributed to inhibit colony spreading.

We then examined whether the *psm-mec* mRNA acts as a regulatory RNA to inhibit colony spreading, PSMα production, and stimulate biofilm formation. By performing a primer extension analysis (Fig. S3), we determined the transcription start site of messenger RNA encoding the *psm-mec* ORF, indicated by red letters in Fig. 1A. In addition to pM1, we constructed pM2 harboring 6 nucleotide substitutions from −15 to −10 of the transcription start site (Fig. 1A). The *psm-mec* mRNA and PSM-mec protein were not detected in the pM2-transformed Newman strain (Fig. 1B, F), indicating that the *psm-mec* ORF promoter was disrupted in pM2. The Newman strain transformed with pM1 or pM2 did not exhibit decreased colony spreading ability, whereas the pF-transformed Newman did (Fig. 1C). Moreover, the pM1- or pM2-transformed Newman strain did not exhibit decreased PSMα production (α3, Fig. 1E; Hld+α1, Fig. S1B) and did not show enhanced biofilm formation (Fig. 1D). Newman strain transformed with pC1, pC2, or pC3 showed decreased PSMα production, although the Newman strain transformed with pM1 or pM2 did not show decreased PSMα production (Fig. 1E and S1B), indicating that it is not the translation product but the transcription product of the *psm-mec* ORF that acted as a regulatory RNA molecule contribute to inhibit PSMα production.

Newman strains transformed with pC1, pC2, or pC3 did not completely lose their colony spreading inhibitory activity (Fig. 1C) or biofilm formation ability (Fig. 1D), whereas Newman strains transformed with pM1 and pM2 completely lost these activities. These results suggest that not only the translation product but also the transcription product of the *psm-mec* ORF, as a regulatory RNA molecule, contribute to inhibit colony spreading and stimulate biofilm formation.

**Figure 7:**
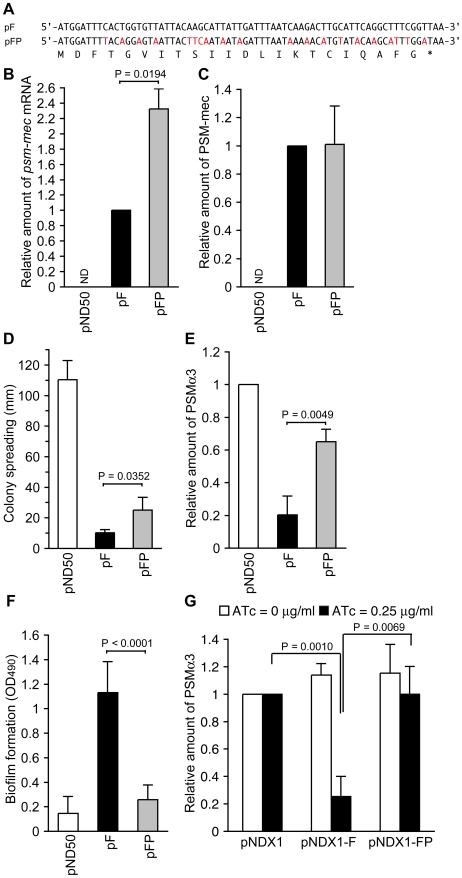
Analysis of synonymous codon substitutions in the *psm-mec* ORF. (A) The nucleotide sequence of the *psm-mec* ORF in pF and the synonymous codon substituted sequence of the *psm-mec* ORF in pFP are shown. The substituted nucleotides are colored in red. The amino acid sequence of PSM-mec protein is shown below the nucleotide sequence. (B) The amounts of *psm-mec* mRNA in Newman strains harboring pND50, pF, and pFP were measured. The data are presented as the means ± standard deviations from at least three independent experiments. ND, not detected. (C) The PSM-mec production of Newman strains harboring pND50, pF, or pFP was examined by HPLC. The data are presented as the means ± standard deviations from at least three independent experiments. ND, not detected. (D) The colony-spreading abilities of Newman strains harboring pND50, pF, or pFP were examined. Plates were incubated for 8 h at 37°C and the means ± standard deviations of the halo diameters from at least three independent experiments are shown. (E) The PSMα production of Newman strains harboring pND50, pF, or pFP was examined by HPLC. The data are presented as the means ± standard deviations from at least three independent experiments. (F) Biofilm formation onto polystyrene microplates of Newman strains harboring pND50, pF, or pFP was examined. (G) The PSMα production of Newman strains harboring the *psm-mec* gene-inducible plasmid (pNDX1-F) was examined. pNDX1-FP harbors the synonymous codon-substituted sequence of the *psm-mec* ORF in (A). ATc, anhydrotetracycline.

To further examine whether the *psm-mec* ORF transcript functions as a regulatory RNA molecule, we constructed pFP, which harbors synonymous codon substitutions in the *psm-mec* ORF (Fig. 7A). The mutated *psm-mec* ORF harbors 20 nucleotide substitutions within 69 bases of the *psm-mec* ORF, changing the secondary structure of its mRNA (data not shown), as estimated by the M. Zuker Mfold program (http://mfold.bioinfo.rpi.edu/cgi-bin/rna-form1.cgi) [31]. The pFP-transformed Newman strain produced approximately twice the amount of *psm-mec* mRNA and nearly the same amount of PSM-mec as the pF-transformed Newman strain (Fig. 7B, C). The pFP-transformed Newman strain showed higher colony-spreading ability (Fig. 7D) and produced more PSMα than the pF-transformed Newman strain (α3, Fig. 7E; Hld+α1, Fig. S1C). Moreover, the pFP-transformed Newman strain formed little biofilm compared with the pF-transformed Newman strain (Fig. 7F). Thus, pFP, which harbors synonymous codon substitutions in the *psm-mec* ORF, showed decreased inhibition of colony-spreading, PSMα production, and stimulation of biofilm formation. To further address whether the *psm-mec* ORF transcript contributes to inhibit PSMα production, we placed the *psm-mec* ORF under an anhydrotetracycline-inducible promoter. Induction of *psm-mec* transcription with anhydrotetracycline decreased PSMα production, whereas induction of the synonymous codon-substituted *psm-mec* did not inhibit PSMα production (Fig. 7G). These results also suggest that the *psm-mec* ORF transcript functioned as a regulatory RNA for these phenomena.

Hfq is an RNA chaperone that mediates the interaction between small RNA and mRNA. *S. aureus hfq* has a global regulatory role for virulence genes in the NCTC8325-4 strain [32] but not in the Newman strain [33]. To verify whether the *hfq* gene is required for the effect of the *psm-mec* ORF on colony spreading, PSMα production, and biofilm formation, we constructed an *hfq*-deleted mutant of Newman and NCTC8325-4 that were transformed with pF. pF inhibited colony spreading and PSMα production, whereas it increased biofilm formation in the *hfq-*deleted mutant of the Newman strain as well as in the Newman strain (Fig. S4A, B, C). pF also inhibited PSMα1 + Hld production, whereas it increased biofilm formation in the *hfq-*deleted mutant of NCTC8325-4 as well as in NCTC8325-4 (Fig. S4B, C). These results suggest that the *psm-mec* ORF inhibits colony spreading and PSMα production, whereas it increases biofilm formation in an *hfq*-independent manner.

## Discussion

**Figure 8:**
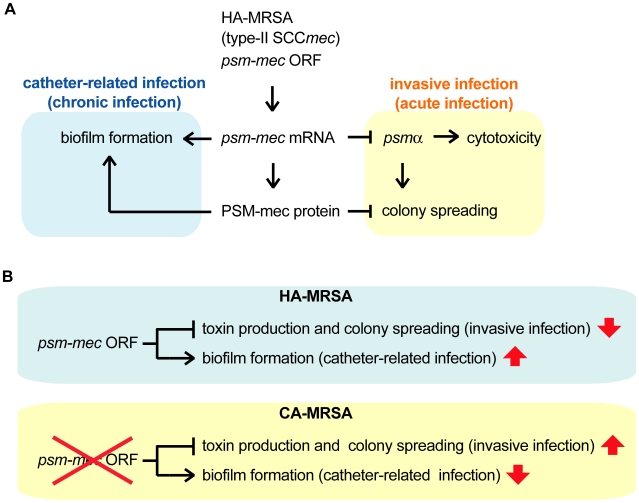
Model of the alteration of *S. aureus* virulence phenotype exerted by *psm-mec* mRNA and PSM-mec protein. (A) The transcript of the *psm-mec* ORF inhibits the expression of *psmα*, contributing to the decreased colony spreading and decreased cytotoxicity. The translation product of the *psm-mec* ORF inhibits colony spreading, whereas it promotes biofilm formation. These altered phenotypes have decreased *S. aureus* virulence, which leads to invasive and acute infections, whereas increased *S. aureus* virulence leads to chronic infection, including catheter-related infections. (B) In CA-MRSA strains, absence of the *psm-mec* ORF leads to increased virulence, causing invasive infections, whereas decreased virulence causes catheter-related infections.

In the present study, we found that the translation product as well as the transcription product of the *psm-mec* ORF in the F region suppresses colony spreading and promotes biofilm formation in *S. aureus* (Fig. 8A). We also revealed that the transcription product of the *psm-mec* ORF decrease the production of PSMα, which is core genome-encoded [12], [17] (Fig. 8A). We previously reported that introduction of the F region into the Newman strain decreases its virulence in a mouse systemic infection model [15]. In the present study, introducing the F region into MW2 and FRP3757, which are CA-MRSA strains, also decreased their virulence in a mouse systemic infection model. Thus, the absence of the *psm-mec* ORF in CA-MRSA strains, a distinguishable feature from HA-MRSA strains harboring type-II SCC*mec*, restores PSMα production and contributes to the high virulence phenotype (Fig. 8A, B).

We assume that the attenuated virulence of the F region-transformed *S. aureus* strains in a mouse systemic infection model is caused by a decrease in both colony spreading and PSMα production. The decreased colony-spreading ability might lead to defective *S. aureus* dissemination into various organs in the animal body, resulting in attenuated virulence (Fig. 8A). It is not clear, however, whether colony spreading is directly involved in the *S. aureus* virulence in animals. Further experiments are needed to address this point.

Queck *et al.* reported that the *psm-mec*-deleted mutant of the MSA890 strain, in which the production of PSM-mec is higher than other genome-encoded PSMs, showed attenuated virulence in a mouse systemic infection model and decreased cytolytic activity against neutrophils [17]. Whereas the *psm-mec*-deleted strains of *S. aureus*, in which PSM-mec production is lower than other genome-encoded PSMs, did not show decreased cytolytic activity against neutrophils [17]. Based on these observations, Queck *et al.* proposed that PSM-mec has a positive effect on the virulence of *S. aureus* strains, in which a higher amount of PSM-mec is produced compared to other genome-encoded PSMs [17]. On the other hand, we demonstrated that introduction of the *psm-mec* ORF into any of the Newman, MW2, and FRP3757 strains has negative effects on virulence in a mouse systemic infection model. According to the proposal by Queck *et al.*, expression of PSM-mec is expected to be much lower than PSMαs in these strains. The difference in the genetic backgrounds of *S. aureus*, for example mutations in the promoters of *psm-mec* and *psmα*, might affect the ratio of the expression levels of PSM-mec and PSMαs. If too much PSM-mec is produced, the positive effect of PSM-mec on virulence may be dominant.

We demonstrated that introducing the *psm-mec* ORF into Newman, MW2, and FRP3757 strains increases biofilm formation. Biofilm formation by *S. aureus* is considered to be important for catheter-related infections [26], [34], [35], [36]. Thus, the *psm-mec* ORF is postulated to have positive effects on catheter-related *S. aureus* infections, which are associated with biofilm formation (Fig. 8A). Using a *psm-mec-*deleted mutant of the MSA890 strain, Otto *et al.* also demonstrated that the *psm-mec* gene stimulates biofilm formation [17]. Therefore, the *psm-mec* ORF in HA-MRSA inhibits the virulence properties that lead to invasive infections accompanied by PSMα production and colony spreading, whereas it promotes the virulence properties for chronic infections such as catheter-related infections (Fig. 8A, B). Thus, the *psm-mec* ORF may regulate the virulence property of *S. aureus*. The function of the *psm-mec* ORF may be beneficial for HA-MRSA to establish long-lasting infection in the human body. In contrast, in CA-MRSA strains, the absence of regulation by the *psm-mec* ORF leads to increased virulence properties that cause invasive and acute infections in humans (Fig. 8B). Our proposed mechanism may explain a number of observations that CA-MRSA causes more severe invasive infections, such as hemorrhagic necrotizing pneumonia, septicemia, and necrotizing fasciitis, than HA-MRSA [37], [38], [39], [40], [41], [42]. In addition, the mutation (−7T>C) in the *psm-mec* promoter that decreases the amount of *psm-mec* mRNA is found in 25% of HA-MRSA strains isolated in Japan [15]. These strains might have different virulence properties compared with most Japanese HA-MRSA strains carrying the intact *psm-mec* promoter. Future studies with *psm-mec*-deleted mutants should address whether endogenous *psm-mec* regulates the virulence of HA-MRSA strains or whether HA-MRSA strains have already adapted to the presence of *psm-mec*. HA-MRSA strains do not necessarily possess the *psm-mec* ORF. MRSA strains having type-I SCC*mec* or type-IV SCC*mec*, which do not carry the *psm-mec* ORF, have been isolated from hospitals in European countries and Australia [43], [44]. Further studies are needed to determine whether the absence of the *psm-mec* in these HA-MRSA strains affects the virulence properties.

We propose that both the transcription product and translation product of the *psm-mec* ORF, which is a cytolytic peptide gene encoded on the mobile genetic element SCC*mec*, alter the virulence properties of the pathogen. To our knowledge, this is the first report that a transcription product of a toxin gene, acting as a regulatory RNA that is encoded on the mobile genetic element, suppresses the expression of core genome-encoded toxin genes. RNAIII is one of the regulatory RNAs in *S. aureus* that is encoded in the *agr* locus and regulates the expression of various virulence genes [21]. RNAIII contains an ORF encoding Hld, a PSM [45]. Thus, both RNAIII and the *psm-mec* transcript encode PSM. Further studies are needed to determine how the *psm-mec* transcript exerts its regulatory function as an RNA. The inhibitory effect of *psm-mec* on PSMα production is probably not due to a direct interaction between *psm-mec* RNA and *psmα* mRNA, but rather to an interference of the transcriptional regulatory pathways of the *psmα* operon, because the promoter activity of the *psmα* operon was inhibited by introduction of the *psm-mec* (Fig. 3F). The SCC*mec* region containing the *psm-mec* ORF is also found in *S. epidermidis* [15]. The *psm-mec* ORF stimulated biofilm formation in *S. epidermidis* (Fig. 5E). The regulation of virulence properties by both transcript and translation products of the *psm-mec* ORF may not be specific for *S. aureus*, but are presumably conserved among pathogens carrying the *psm-mec* ORF on the mobile genetic element SCC*mec*.

## Materials and Methods

### Ethics statement

This study was carried out in strict accordance with the recommendation in the Fundamental Guidelines for Proper Conduct of Animal Experiment and Related Activities in Academic Research Institutions under the jurisdiction of the Ministry of Education, Culture, Sports, Science and Technology, 2006. All mouse protocols followed the Regulations for Animal Care and Use of the University of Tokyo and were approved by the Animal Use Committee at the Graduate School of Pharmaceutical Science at the University of Tokyo (approval number: 19–28).

### Bacterial strains and growth conditions

The JM109 strain of *Escherichia coli* was used as the host for pND50, pKOR3a, and pSF151, and their derivatives. *E. coli* strains transformed with the plasmids were cultured in Luria-Bertani broth containing 25 µg/ml chloramphenicol or 50 µg/ml kanamycin. *S. aureus* strains were aerobically cultured in tryptic soy broth at 37°C in a 50-ml disposable tube (FALCON 352070, Becton, Franklin Lakes, NJ), and 12.5 µg/ml chloramphenicol or 50 µg/ml kanamycin was added to the medium if required. Details of the bacterial strains and plasmids used in the present study are shown in Table 1.

**Table 1:** A list of bacterial strains and plasmids used.

Strain or plasmid	Genotypes or characteristics^a^	Source or reference
Strains		
*S. aureus*		
RN4220	NCTC8325-4, restriction mutant	[52]
Newman	Laboratory strain, High level of clumping factor	[53]
NCTC8325-4	NCTC8325 cured of ϕ11, ϕ12, and ϕ13	[54]
N315	methicillin resistant	[55]
YS1	Newman Δ*psmα*::*ermAM*	This study
YS2	Newman Δ*psmβ*::*aph*	This study
YS3	Newman Δ*hfq*::*aph*	This study
YS4	NCTC8325-4 Δ*hfq*::*aph*	This study
GN1	Newman Δ*fnbA*::pT2291	This study
MW2	CA-MRSA, USA400	[56]
FRP3757	CA-MRSA, USA300	[57]
*S. epidermidis*		
ATCC12228	A non-biofilm forming strain	ATCC
*E. coli*		
JM109	General purpose host strain for cloning	Takara Bio
Plasmids		
pKOR3a	Vector for alleic replacement in *S. aureus*, Cm^r^	[58]
pND50	*E. coli*-*S. aureus* shuttle vector; Cm^r^	[59]
pF	pND50 with intact *fudoh* and *psm-mec* from N315	[15]
pM1	pND50 with deficient promoter of *psm-mec*	[15]
pM2	pND50 with deficient promoter of *psm-mec*	This study
pB1	pND50 with Y27 Stop *fudoh* and intact *psm-mec*	This study
pB2	pND50 with K36 Stop *fudoh* and intact *psm-mec*	This study
pB3	pND50 with Y45 Stop *fudoh* and intact *psm-mec*	This study
pB4	pND50 with K52 Stop *fudoh* and intact *psm-mec*	This study
pC1	pND50 with K16 Stop *fudoh* and F3 Stop *psm-mec*	This study
pC2	pND50 with V11 Stop *fudoh* and T8 Stop *psm-mec*	This study
pC3	pND50 with K6 Stop *fudoh* and L13 Stop *psm-mec*	This study
pFP	pND50 with codon-replaced *psm-mec* ORF	This study
pluc	pND50 with *luc+* with a ribosomal binding site	[50]
pluc-F	pluc with *psm-mec* from N315	This study
pluc-psmαP	pluc with *psmα* promoter	This study
pluc-psmαP-F	pluc-F with *psmα* promoter	This study
pND50K	*E. coli*-*S. aureus* shuttle vector; Kan^r^	This study
ppsmα	pND50K with *psmα* operon from Newman	This study
pCK20	*E. coli* vector; Cm^r^	[60]
pInt	pCK20 with partial genomic region from RN4220	[15]
pIntF	pInt with intact *fudoh* and *psm-mec* from N315	[15]
pSF151	*E. coli* vector; Kan^r^	[61]
pT2291	pSF151 with internal *fnbA* from Newman	This study
pNDX1	pND50 carrying TetR and *xyl/tet* from pWH353	[62]
pNDX1-F	pNDX1 with 5′-UTR, *psm-mec* ORF, and 3′-UTR	This study
pNDX1-FP	pNDX1 with 5′-UTR, codon-replaced *psm-mec* ORF, and 3′-UTR	This study

a Cm, chloramphenicol; Erm, erythromycin; Kan, kanamycin.

### Mouse infection experiment

Bacterial overnight cultures were centrifuged and cells were suspended in phosphate buffered saline. The bacterial suspension (100 µl) was injected into the tail vein of 8-week-old female CD-1 mice. Survival after the injection was monitored.

### Colony spreading assay

Tryptic soy broth (Becton, Sparks, MD) supplemented with 0.24% agar (Code 01028-14, Nacalai Tesque Inc., Kyoto, Japan) was autoclaved at 121°C for 15 min. Sterile medium (50 ml) was poured into a petri dish (150-mm diameter, FALCON 351058, Becton). The plates were dried for 20 min in a biologic safety cabinet (MHE-130AJ, SANYO, Tokyo, Japan). Bacterial overnight culture (2 µl) was spotted onto the center of the plates and dried for 20 min in a biologic safety cabinet. The plates were covered and incubated at 37°C.

### Biofilm formation assay

Four microliters of bacterial overnight culture were inoculated into 1 ml tryptic soy broth containing 0.25% glucose. An aliquot (200 µl) of the sample was poured into each well of a 96-well polystyrene microplate (3860-096, IWAKI, Tokyo, Japan), and incubated for 3 days at 37°C. The cultures in the plate were discarded and the plate was stained with 0.1% safranin solution. The OD_490_ was measured using a microplate reader (MTP300, CORONA, Ibaraki, Japan). To observe the biofilm formation on polypropylene, bacterial colonies were inoculated into 5 ml of tryptic soy broth and aerobically cultured for 3 days in 50-ml tubes (352070, Becton Dickinson, Franklin Lakes, NJ) at 37°C.

### Measurement of PSMs

Overnight bacterial cultures (50 µl) were inoculated into 5 ml fresh tryptic soy broth and aerobically cultured at 37°C for 14 h without antibiotics. The cultures were filtered with a 0.22-µm polyvinylidene difluoride filter (Millipore, Carrigtwohill, Ireland) and the filtrates were used for analysis by reversed phase-HPLC. Chromatography was performed using SOURCE 5RPC ST 4.6/150 column (GE Healthcare, Tokyo, Japan) and a water/acetonitrile gradient in 0.1% trifluoroacetic acid from 0 to 100% acetonitrile in 50 min at a flow rate of 1 ml/min (600E, Waters, Milford, MA). Absorbance at 215 nm was detected using a 2998 Photodiode Array Detector (Waters). The molecular mass in the respective peak was determined using liquid chromatography-electrospray ionization mass spectrometry (LC/ESI-MS; LC 1100 series, Agilent Technologies, Santa Clara, CA; ESI-MS, Bio-TOFQ, Bruker Daltonics, Billerica, MA) and respective PSMs were identified (Fig. S2). Although there was a difference in the retention time between chromatographies in the LC/MS and HPLC systems (around 4 min faster in the LC/ESI-MS system), the pattern of the respective PSMs was similar. Hld and PSMα1 were not separated in both systems.

### DNA manipulation

Transformation of *E. coli*, extraction of plasmid DNA from *E. coli*, and PCR were performed as previously described [46]. *S. aureus* genomic DNA was extracted using a QIAamp DNA Blood Kit (Qiagen Sciences, Germantown, MD) and lysostaphin (Takara Bio). Transformation of *S. aureus* with plasmid DNA was performed by electroporation [47].

### Determination of the transcriptional start site of the *psm-mec* ORF

Oligonucleotide primer 5AA-F was end-labeled with [γ-^32^P] ATP using T4 polynucleotide kinase. RNA was reverse-transcribed using the labeled primer and Multiscribe Reverse Transcriptase (Roche, Basel, Switzerland). Sequencing ladder samples were obtained by cycle-sequencing reactions using the labeled primer, DNA fragments of the F region, and Thermo sequencing primer cycle sequencing kit (GE Healthcare). The samples were electrophoresed in a denaturing 7.5% polyacrylamide gel containing 6 M urea in 0.5×TBE buffer [45 mM Tris borate (pH8.3), 1 mM Na_2_EDTA]. The gels were dried and analyzed by phosphoimaging using BAS-1800II (Fujifilm, Tokyo, Japan) and Image Gauge software v. 4.23 (Fujifilm).

### Construction of gene-disrupted mutants for the *psmα* operon, the *psmβ* operon, the *fnbA* gene, and the *hfq* gene

The upstream region of the *psmα* operon (966 bp) was amplified by PCR using oligonucleotide primers psma-U-F and psma-U-R, and Newman genomic DNA as the template. The downstream region of the *psmα* operon (979 bp) was amplified by PCR using oligonucleotide primers psma-D-F and psma-D-R, and Newman genomic DNA as the template. The *ermAM* gene, conferring erythromycin resistance, was amplified by PCR using oligonucleotide primers ErmF and ErmR, and pMutinT3 as the template. These three DNA fragments were spliced together using splicing by overlap extension-PCR, resulting in a psmα-cassette. The psmα-cassette was inserted into the *Sma* I site of pKOR3a, resulting in pKOR3a-psmα. *S. aureus* RN4220 was transformed with pKOR3a-psmα. The transformant was cultured in tryptic soy broth and 10^3^ cells were spread onto tryptic soy agar plates containing 12.5 µg/ml chloramphenicol. The plates were incubated at 43°C overnight. The resulting colonies were cultured in tryptic soy broth at 37°C and spread onto tryptic soy agar plates containing 1 µg/ml anhydrotetracycline and 10 µg/ml erythromycin. The resulting colonies were examined for sensitivity to chloramphenicol. The disruption was transferred to the Newman strain by phage 80α, as reported previously [48], resulting in YS1. The deletion of *psmα* was confirmed by Southern blot analysis (Fig. S5A, B).

To construct the *psmβ*-deleted mutant, primers of psmb-U-F, psmb-U-R, psmb-D-F, and psmb-D-R were used for to amplify the upstream (1546 bp) and downstream (1727bp) regions of the *psmβ* operon and primers of KanF and KanR were used to amplify the kanamycin resistance encoding gene, *aph*, from pSF151. The amplified DNA fragments were spliced together by splicing by overlap extension-PCR, resulting in a psmβ-cassette. Other procedures were the same with as that for the *psmα*-deleted mutant. Disruption of *psmβ* in the YS2 strain was confirmed by Southern blot analysis (Fig. S5A, C).

To construct the *fnbA*-disrupted mutant, the internal region of *fnbA* was amplified by PCR using oligonucleotide primers fnbA-F and fnbA-R, and Newman genomic DNA as the template. The amplified DNA fragment was inserted into pSF151, resulting in pT2291. *S. aureus* RN4220 was transformed with pT2291 and kanamycin-resistant transformants were obtained. The disruption was transferred to the Newman strain by phage 80α, resulting in GN1. The disruption of *fnbA* was confirmed by Southern blot analysis (Fig. S5D, E).

To construct the *hfq-*deleted mutant, the upstream (886 bp) and downstream (821 bp) regions of the *hfq* gene and *aph* gene were amplified by PCR and spliced together by overlap extension-PCR, resulting in an hfq-cassette. Other procedures were the same as that used for the *psmα*-deleted mutant. Disruption of *hfq* in the YS3 and YS4 strains was confirmed by Southern blot analysis (Fig. S5F, G).

### Construction of plasmids harboring shortened F-regions, point-mutated F-regions, or *psm-mec* with inducible promoter

To construct plasmids harboring a shortened F-region, we amplified DNA fragments by PCR using the primers listed in Table S2, pF as a template, and KOD-Plus DNA polymerase (TOYOBO, Tokyo, Japan). The amplified DNA fragments were self-ligated, which resulted in the plasmids harboring the shortened F-region. The desired constructs of the plasmids were confirmed by restriction digestion and sequencing. To construct plasmids harboring point-mutated F-regions, we synthesized mutated DNA strands by thermal cycling using primer pairs in Table S2 and pF as a template. The *E. coli* JM109 strain was transformed with the synthesized DNA strands after treatment with *Dpn* I [49]. The plasmids were extracted and sequenced to confirm the desired mutation. To construct pFP, we performed three rounds of nested PCR using primer pairs of onlyP-F and onlyP-R, onlyP-F1 and onlyP-R1, or onlyP-F2 and onlyP-R1 (Table S2), and pF as a template. *E. coli* JM109 strain was transformed with the amplified DNA fragments. The plasmids were extracted and sequenced to confirm the desired mutation. To construct plasmid harboring *psm-mec* with *xyl/tet* promoter, 575 bp F-region was cloned into *Sma* I site of pNDX1 and the upstream region of the transcription start site of *psm-mec* was removed by PCR using the primers listed in Table S2. The transcription start site of *psm-mec* from pNDX1-F was confirmed to be same with that from pF by primer extension analyses (data not shown).

### Measurement of gene expression by quantitative real-time PCR analysis

RNA was extracted from exponentially growing *S. aureus* cells (A_600_ = 1) using an RNeasy Mini Kit (Qiagen, Gaithersburg, MD). RNA was reverse-transcribed to cDNA using Multiscribe Reverse Transcriptase (Roche). Quantitative real-time PCR was performed using cDNA as template and SYBR Premix ExTaq (Takara Bio, Tokyo, Japan) and primers (Table S2). The signals were detected by ABI PRISM 7700 Sequence Detector (Applied Biosystems, Tokyo, Japan). The reaction mixture was incubated at 95°C for 10 s and at 40 cycles (95°C, 5 s; 60°C, 31 s). The data were normalized to 16S rRNA. To determine the amount of the *psm-mec* mRNA in Fig. 7, we used primer pairs of psm-mecF2 and psm-mecR2, which hybridized with the outside region of *psm-mec* ORF. The amplification efficiency was not different between pF and pFP.

### Reporter assay

Pluc was designed to contain a functional ribosomal binding site and translational start codon of *luc* after a series of stop codons in all reading frames [50]. DNA fragment containing the F region was inserted into *Eco*R I and *Sac* I site of pluc vector, resulting in pluc-F harboring the *psm-mec* ORF that was transcribed in the opposite direction from the *luc* ORF. The DNA fragment containing the promoter region of the *psmα* operon [22] was amplified by PCR and inserted into the *Kpn* I and *Xba* I sites of pluc and pluc-F. The staphylococcal strains transformed with pluc, pluc-F, and their derivatives were cultured and harvested at A_600_ = 1. The cells were lysed in a lysis buffer (25 mM KH_2_PO_4_ [pH 7.8], 0.04% Triton X-100, 0.1 mM dithiothreitol, 10 µg/ml of lysostaphin, and protease inhibitor cocktail [Roche, Basel, Switzerland]). The supernatant of the cell lysate was incubated with the luciferase substrate (Roche), and luminescence was measured using a luminometer (Berthold Technologies, Bad WildBad, Germany). The promoter activity was calculated as the luminescence unit per milligram of protein subtracted from the value of the cells transformed with the vector, pluc, or pluc-F.

## Supporting Information

Figure S1 The amount of PSMα1+Hld in Newman strains transformed with various plasmids carrying a mutated F region. The amount of PSMα1+Hld was measured by HPLC. (A), Newman strain transformed with plasmids carrying domain deletions of the F region; (B), Newman strains transformed with plasmids carrying the nucleotide substituted F region; (C), Newman strain transformed with pFP carrying the synonymous codon substituted *psm-mec* ORF.
(1.10 MB TIF)

Figure S2 Determination of PSM species by TOF/MS. (A) Overnight culture of a Newman strain harboring pND50 was fractionated with LC/ESI-MS (Bio-TOFQ, Bruker). Chromatography was performed using SOURCE 5RPC ST 4.6/150 column (GE Healthcare, Tokyo, Japan) and a water/acetonitrile gradient in 0.1% trifluoroacetic acid from 0 to 100% acetonitrile in 50 min at a flow rate of 1 ml/min. (B) Detected *m/z* from peaks 1, 2 3, and 4 in (A) are presented. The highest peaks are the first 13C isotope peaks. Predicted monoisotopic *m/z* for respective PSMs are follows; N-formylated PSMα1 (the monoisotopic molecular weight [MW], 2286.34), 1144.2 [M + 2H]^2+^ and 763.11 [M + 3H]^3+^; N-formylated PSMα2 (MW, 2304.37), 1153.2 [M + 2H]^2+^ and 769.12 [M + 3H]^3+^; N-formylated PSMα3 (MW, 2633.41), 1317.7 [M + 2H]^2+^ and 878.80 [M + 3H]^3+^; N-formylated PSMα4 (MW, 2198.35), 1100.2 [M + 2H]^2+^ and 733.78 [M + 3H]^3+^; N-formylated PSM-mec (MW, 2413.23), 1207.6 [M + 2H]^2+^; N-formylated Hld (MW, 3004.6), 1503.3 [M + 2H]^2+^, 1002.5 [M + 3H]^3+^, and 752.16 [M + 4H]^4+^. A comparison of the detected *m/z* with the predicted *m/z* indicated that peak 1 contains PSMα3; peak2 contains PSMα2; peak3 contains Hld and PSMα1; peak 4 contains PSMα4. For PSM-mec, we analyzed the culture supernatant of Newman strain harboring pF and observed that a peak at 37.8–38.2 min gives 1208.34 *m/z* (data not shown).
(1.21 MB TIF)

Figure S3 Determination of the transcription start site for the *psm-mec* ORF. RNA was extracted from Newman harboring pND50 (lanes 3 and 4) or pF (lanes 1 and 2) and was used as template for reverse transcription with primer 5AA-F (Table S2). Lanes 1 and 3, presence of reverse transcriptase; lanes 2 and 4, absence of reverse transcriptase. A, C, G, and T indicate a sequencing ladder. Asterisk corresponds to the migration of the band in lane 1.
(2.29 MB TIF)

Figure S4 The *psm-mec* exerts its effect in an *hfq-*independent manner. Newman harboring pND50 or pF; the *hfq-*deleted Newman strain (YS3) harboring pND50 or pF, NCTC8325-4 harboring pND50 or pF; and the *hfq-*deleted NCTC8325-4 (YS4) harboring pND50 or pF were examined for colony spreading (A), PSMα production (B), and biofilm formation on polystyrene microplates (C). The data are presented as the means ± standard deviations from at least three independent experiments.
(1.08 MB TIF)

Figure S5 Construction of the mutants for *psmα*, *psmβ*, *fnbA*, and *hfq*. (A) Southern blot analysis of the *psmα*- and *psmβ*-deleted mutants. Lanes 1 and 7, Newman; lanes 2, 3, 4, and 5, *psmα*-deleted mutant (YS1); lanes 8, 9, 10, 11, 12, and 13, *psmβ*-deleted mutant. (B) Restriction maps around the *psmα* operon in the Newman strain and the *psmα*-deleted mutant are presented. (C) Restriction maps around the *psmβ* operon in the Newman strain and the *psmβ*-deleted mutant are presented. (D) Southern blot analysis of the *fnbA-*disrupted mutants. Lane 1, Newman; lane 2, 3, and 4, the *fnbA-*disrupted mutant. (E) Restriction maps around the *fnbA* gene in the Newman strain and the *fnbA-*disrupted mutant are presented. (F) Southern blot analysis of the *hfq-*deleted mutants. Lane 1, Newman; lane 2, Newman/pND50; lane 3, Newman/pF; lanes 4, 5, and 6, *hfq-*deleted mutant of Newman (YS3)/pND50; lanes 7, 8, and 9, YS3/pF; lane 10, NCTC8325-4; lanes 11 and 12, NCTC8325-4/pND50; lanes 13, 14, and 15, NCTC8325-4/pF; lanes 16 and 17, *hfq-*deleted mutant of NCTC8325-4 (YS4)/pND50; lanes 18, 19, and 20, YS4/pF. (G) Restriction maps around the *hfq* gene in the Newman and NCTC8325-4 strains and the *hfq-*deleted mutants are presented.
(5.01 MB TIF)

Table S1 Summary of differentially expressed proteins between the F region-introduced Newman strain and the empty vector-introduced Newman strain.
(0.07 MB PDF)

Table S2 PCR primers used in the study.
(0.08 MB PDF)
