# Supplementary figures and images for: Transcription and Translation Products of the Cytolysin Gene psm-mec on the Mobile Genetic Element SCCmec Regulate Staphylococcus aureus Virulence
Source: PLoS Pathog. 2011 Feb 3;7(2):e1001267. doi: 10.1371/journal.ppat.1001267 (PMC3033363; doi:10.1371/journal.ppat.1001267)

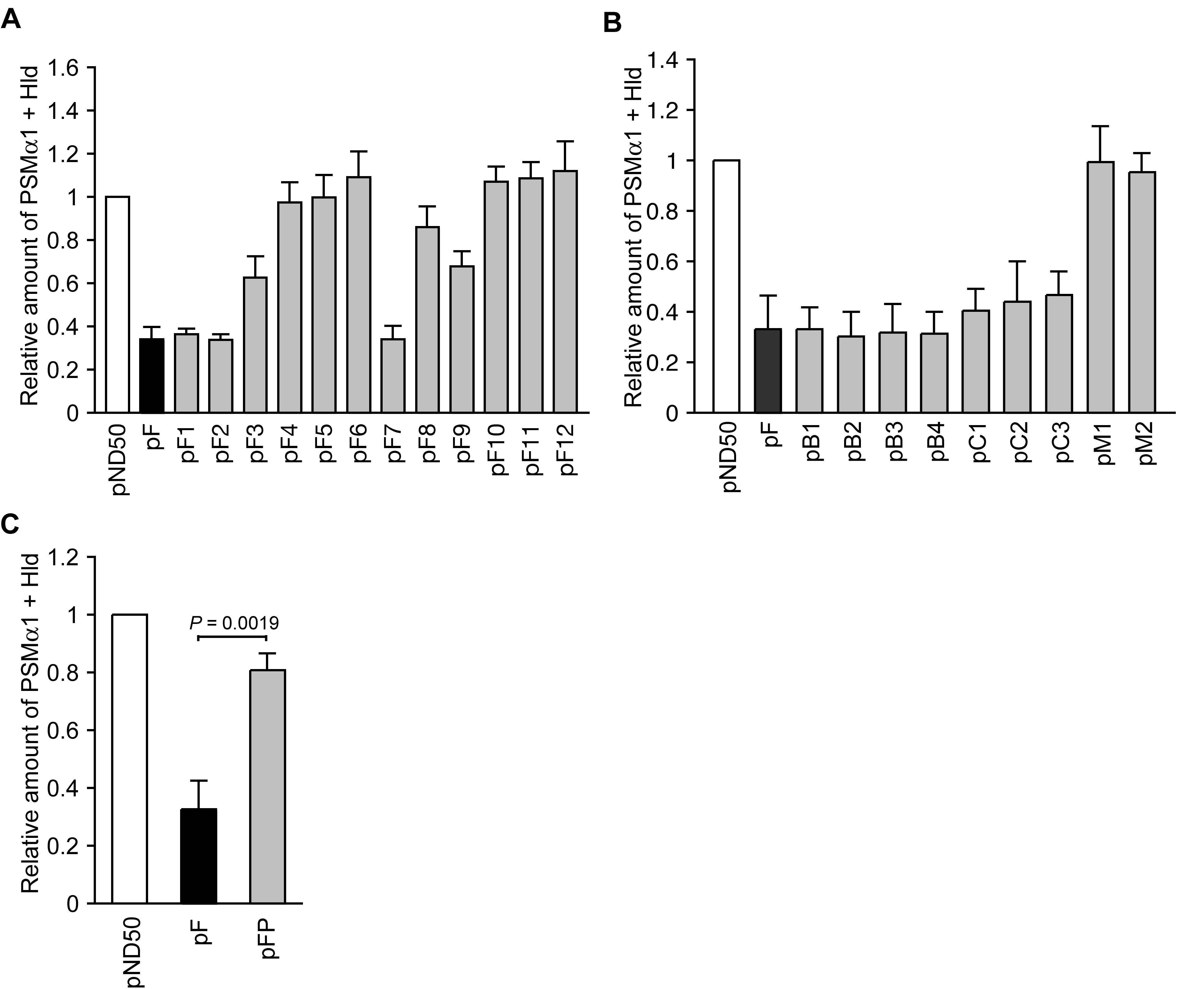

Supplement: Figure S1 — The amount of PSMα1+Hld in Newman strains transformed with various plasmids carrying a mutated F region. The amount of PSMα1+Hld was measured by HPLC. (A), Newman strain transformed with plasmids carrying domain deletions of the F region; (B), Newman strains transformed with plasmids carrying the nucleotide substituted F region; (C), Newman strain transformed with pFP carrying the synonymous codon substituted psm-mec ORF. (1.10 MB TIF) [file ppat.1001267.s001.tif]

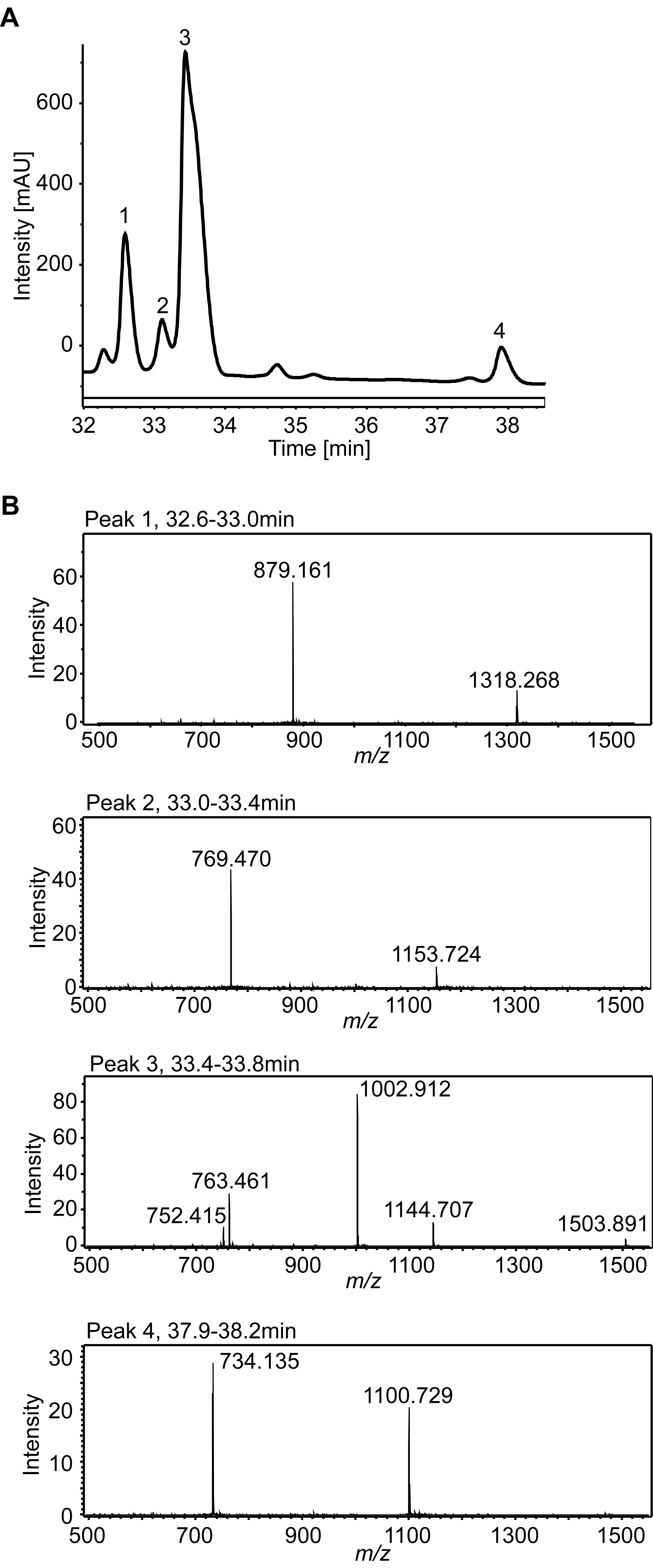

Supplement: Figure S2 — Determination of PSM species by TOF/MS. (A) Overnight culture of a Newman strain harboring pND50 was fractionated with LC/ESI-MS (Bio-TOFQ, Bruker). Chromatography was performed using SOURCE 5RPC ST 4.6/150 column (GE Healthcare, Tokyo, Japan) and a water/acetonitrile gradient in 0.1% trifluoroacetic acid from 0 to 100% acetonitrile in 50 min at a flow rate of 1 ml/min. (B) Detected m/z from peaks 1, 2 3, and 4 in (A) are presented. The highest peaks are the first 13C isotope peaks. Predicted monoisotopic m/z for respective PSMs are follows; N-formylated PSMα1 (the monoisotopic molecular weight [MW], 2286.34), 1144.2 [M + 2H]2+ and 763.11 [M + 3H]3+; N-formylated PSMα2 (MW, 2304.37), 1153.2 [M + 2H]2+ and 769.12 [M + 3H]3+; N-formylated PSMα3 (MW, 2633.41), 1317.7 [M + 2H]2+ and 878.80 [M + 3H]3+; N-formylated PSMα4 (MW, 2198.35), 1100.2 [M + 2H]2+ and 733.78 [M + 3H]3+; N-formylated PSM-mec (MW, 2413.23), 1207.6 [M + 2H]2+; N-formylated Hld (MW, 3004.6), 1503.3 [M + 2H]2+, 1002.5 [M + 3H]3+, and 752.16 [M + 4H]4+. A comparison of the detected m/z with the predicted m/z indicated that peak 1 contains PSMα3; peak2 contains PSMα2; peak3 contains Hld and PSMα1; peak 4 contains PSMα4. For PSM-mec, we analyzed the culture supernatant of Newman strain harboring pF and observed that a peak at 37.8–38.2 min gives 1208.34 m/z (data not shown). (1.21 MB TIF) [file ppat.1001267.s002.tif]

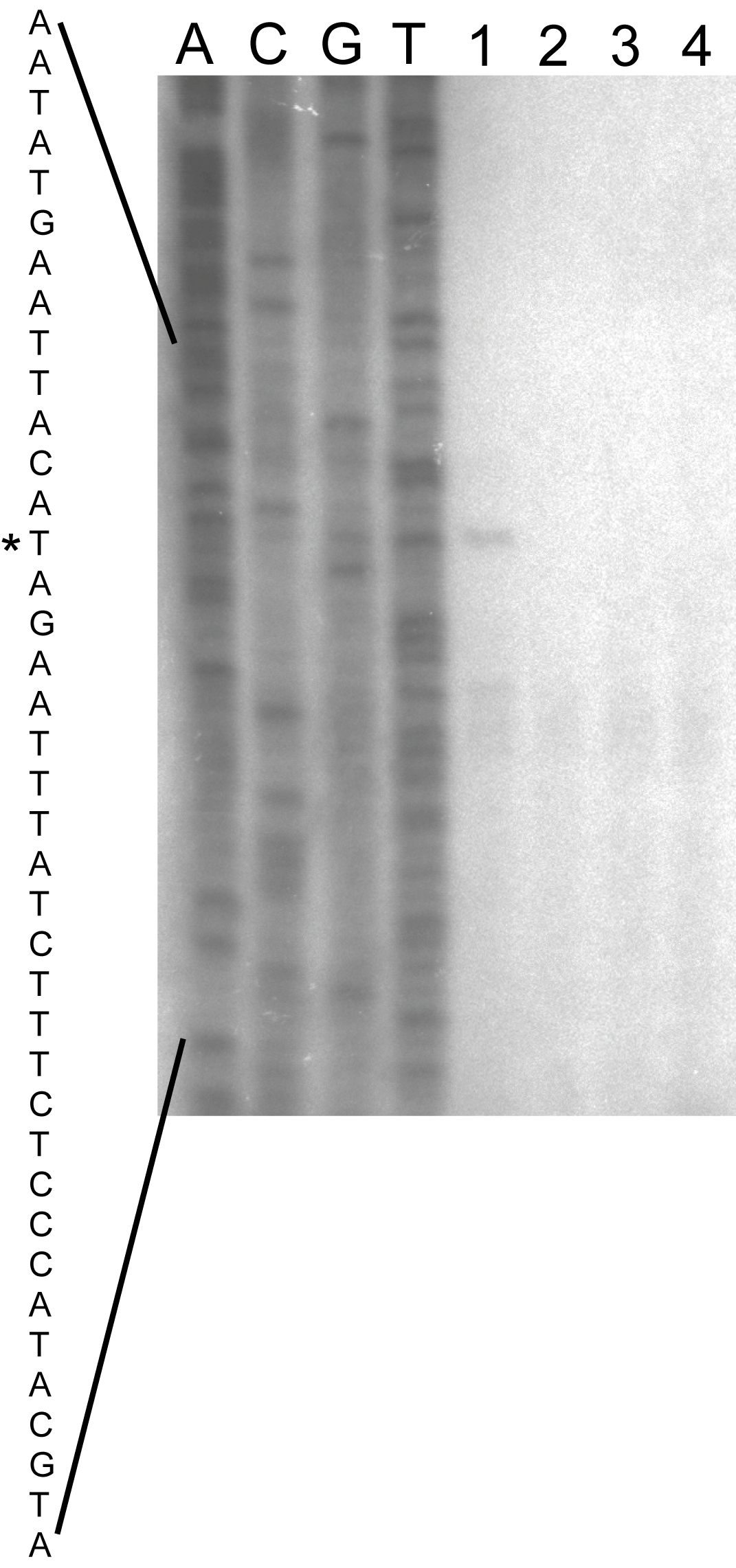

Supplement: Figure S3 — Determination of the transcription start site for the psm-mec ORF. RNA was extracted from Newman harboring pND50 (lanes 3 and 4) or pF (lanes 1 and 2) and was used as template for reverse transcription with primer 5AA-F (Table S2). Lanes 1 and 3, presence of reverse transcriptase; lanes 2 and 4, absence of reverse transcriptase. A, C, G, and T indicate a sequencing ladder. Asterisk corresponds to the migration of the band in lane 1. (2.29 MB TIF) [file ppat.1001267.s003.tif]

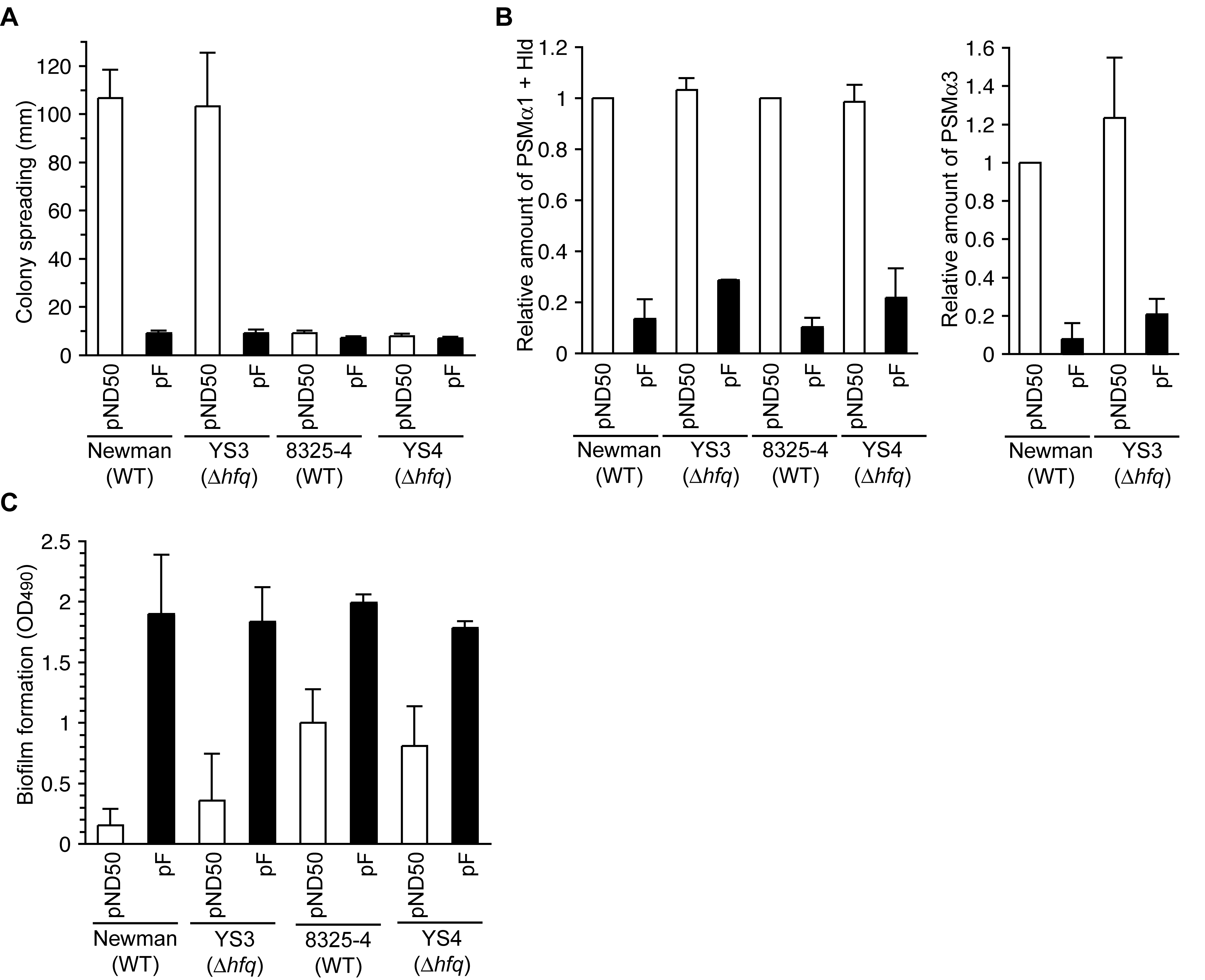

Supplement: Figure S4 — The psm-mec exerts its effect in an hfq-independent manner. Newman harboring pND50 or pF; the hfq-deleted Newman strain (YS3) harboring pND50 or pF, NCTC8325-4 harboring pND50 or pF; and the hfq-deleted NCTC8325-4 (YS4) harboring pND50 or pF were examined for colony spreading (A), PSMα production (B), and biofilm formation on polystyrene microplates (C). The data are presented as the means ± standard deviations from at least three independent experiments. (1.08 MB TIF) [file ppat.1001267.s004.tif]

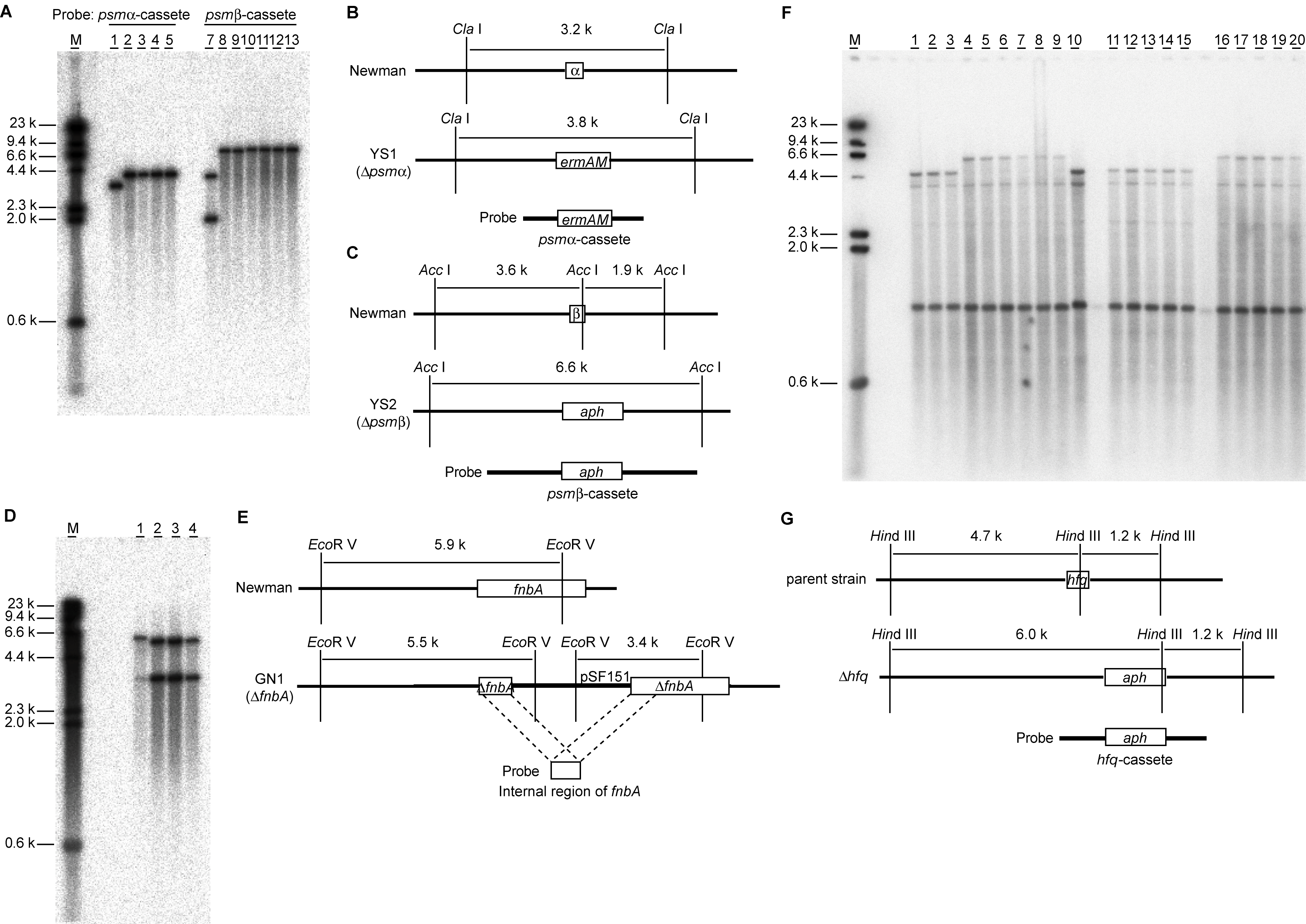

Supplement: Figure S5 — Construction of the mutants for psmα, psmβ, fnbA, and hfq. (A) Southern blot analysis of the psmα- and psmβ-deleted mutants. Lanes 1 and 7, Newman; lanes 2, 3, 4, and 5, psmα-deleted mutant (YS1); lanes 8, 9, 10, 11, 12, and 13, psmβ-deleted mutant. (B) Restriction maps around the psmα operon in the Newman strain and the psmα-deleted mutant are presented. (C) Restriction maps around the psmβ operon in the Newman strain and the psmβ-deleted mutant are presented. (D) Southern blot analysis of the fnbA-disrupted mutants. Lane 1, Newman; lane 2, 3, and 4, the fnbA-disrupted mutant. (E) Restriction maps around the fnbA gene in the Newman strain and the fnbA-disrupted mutant are presented. (F) Southern blot analysis of the hfq-deleted mutants. Lane 1, Newman; lane 2, Newman/pND50; lane 3, Newman/pF; lanes 4, 5, and 6, hfq-deleted mutant of Newman (YS3)/pND50; lanes 7, 8, and 9, YS3/pF; lane 10, NCTC8325-4; lanes 11 and 12, NCTC8325-4/pND50; lanes 13, 14, and 15, NCTC8325-4/pF; lanes 16 and 17, hfq-deleted mutant of NCTC8325-4 (YS4)/pND50; lanes 18, 19, and 20, YS4/pF. (G) Restriction maps around the hfq gene in the Newman and NCTC8325-4 strains and the hfq-deleted mutants are presented. (5.01 MB TIF) [file ppat.1001267.s005.tif]
