# Supplementary material for: Transcription and Translation Products of the Cytolysin Gene psm-mec on the Mobile Genetic Element SCCmec Regulate Staphylococcus aureus Virulence
Source: PLoS Pathog. 2011 Feb 3;7(2):e1001267. doi: 10.1371/journal.ppat.1001267 (PMC3033363; doi:10.1371/journal.ppat.1001267)
Supplement: Table S1 — Summary of differentially expressed proteins between the F region-introduced Newman strain and the empty vector-introduced Newman strain. (0.07 MB PDF) [file ppat.1001267.s006.pdf]

Table S1. Summary of differentially expressed proteins between the F region-introduced Newman strain and the empty vector-introduced Newman strain.

Molecular weight (kDa)		Protein predicted	Peptides mached	Sequence coverage percent
SDS-PAGE	Database			
7*	4.5	hypothetical protein MW1056, PSMβ1 (gi 21282785)	3	91
94	78**	fibronectin binding protein A precursor (gi 50234117)	54	71

The protein band stained with Coomassie brilliant blue was excised and digested in-gel with trypsin. Nanoscale capillary liquid chromatography-tandem mass spectrometry of in-gel digests was performed using a ThermoFisher LTQ Orbitrap XL. Database searching was performed using the Mascot search program (www.matrixscience.com). *The molecular weight of the protein was determined using Tricine-buffered 16.5% sodium dodecyl sulfate polyacrylamide gel electrophoresis. **The molecular weight was estimated from the amino acid sequence of mature FnbA [1].

1. Jonsson K, Signas C, Muller HP, Lindberg M (1991) Two different genes encode fibronectin binding proteins in *Staphylococcus aureus*. The complete nucleotide sequence and characterization of the second gene. Eur J Biochem 202: 1041-1048.
