# Supplementary material for: Transcription and Translation Products of the Cytolysin Gene psm-mec on the Mobile Genetic Element SCCmec Regulate Staphylococcus aureus Virulence
Source: PLoS Pathog. 2011 Feb 3;7(2):e1001267. doi: 10.1371/journal.ppat.1001267 (PMC3033363; doi:10.1371/journal.ppat.1001267)
Supplement: Table S2 — PCR primers used in the study. (0.08 MB PDF) [file ppat.1001267.s007.pdf]

Table S2. PCR primers used in the study.

Purpose or target	Primer	Sequence (5'-3')
plasmid construction	KmF-SpeI	CGGACTAGTCATCCATAGTTGCCTGACTCC
	KmR-XhoI	CCGCTCGAGTCTAGGTACTAAAACAATTCATCCAG
	pND50F-SpeI	CGGACTAGTGGAGGCTTACTTGTCTGCTTTC
	pND50R-XhoI	CCGCTCGAGCCAGATCCGATTGCTGAATAA
primer extension	5AA-F	AAATCAATAATGCTTGTAATAACACCAGTG
aph	KmF	AGCGAACCATTTGAGGTGAT
	KmR	GGGACCCCTATCTAGCGAAC
ermAM	ErmF	TTCGTCTTCAAGAATTGATCCTC
	ErmR	TTCCAAATTTACAAAAGCGACTC
psmα	psma-U-F	ACGTGGCACTTTCCAAAAAC
	psma-U-R	GAGGATCAATTCTTGAAGACGAAGATTACCTCCTTTGCT TATGAGT
	psma-D-F	GAGTCGCTTTTGTAAATTTGGAAATTCTCAGGCCACTATA CCAA
	psma-D-R	TCGTTCGATCAAAAGGTATCG
	psma-C-F	GGAGGATCCAGACACTGCATCACGGTACG
	psma-C-R	GGTGGTACCGGCAAATTAGACCAGCACGA
psmβ	psmb-U-F	GTACCTAAAAAGCCAGACATGAAAA
	psmb-U-R	ATCACCTCAAATGGTTCGCTGCGTAGCTTTTAAATCACCG AAAT
	psmb-D-F	GTTCGCTAGATAGGGGTCCCGTTGCTAACGGTGTGGGTT T
	psmb-D-R	GAAAAGCCCTGCTTGTTGTC
fnbA	fnbA-F	AAGAAGCTTGCTGCAGCATCAGAACAAAA
	fnbA-R	GGAGGATCCCGTTGTCCTGCATGAGGTT
hfq	hfq-U-F	AACTCGCGAAGCGTATCAAT
	hfq-U-R	ATCACCTCAAATGGTTCGCTTGCAATCATCTGTCGGACTC
	hfq-D-F	GTTCGCTAGATAGGGGTCCCTCATATGGGCACGATTTAA TGA
	hfq-D-R	GAATTTGTTGCTGAGCCATGT
shortening of the F region	F	GGAGGATCCTTACGTATTTCCAATATGACG
	F1	TGACGATTTTTTATGCAAAG
	F2	TTTTTCAAATTTTTGACATTTATGCAATCT
	F3	AAATAGCCAATTAGGGAATTTTTAAC
	F4	TTTTTTATTTGTTTGATATTATACTTAATG
	F5	CTTAATGTATCTTAAATAGAAAGAG
	R7	TCAAACAAATAAAAAATGTTAAAAATTCCC
	R6	TTCTATTTAAGATACATTAAGTATAATATC
	R2	AAGAAGCTTGTAATAACACCAGTGAAATC
	R3	AAGAAGCTTTTTTAGTTGAAAAAATTAA
	R4	AAGAAGCTTGTGTTTACAACAATACATAGA
pM2	M2-F	TAACATTTTTTATTTGTTTGACCCCCCACTTAATGTATCTT AAATAGAA
	M2-R	TTCTATTTAAGATACATTAAGTGGGGGGTCAAACAAATA AAAAATGTTA
pB1	TAA1-F	CATACCCTCTTTCTATTTAAGATAAATTAAGTATAATATC AAACAAATA

	TAA1-R	TATTTGTTTGATATTATACTTAATTTATCTTAAATAGAAAGAGGGTATG
pB2	TAA2-F	ATTAAGTATAATATCAAACAAATATAAAATGTTAAAAATTCCCTAATTG
	TAA2-R	CAATTAGGGAATTTTTAACATTTTATATTTGTTTGATATTATACTTAAT
pB3	TAA3-F	TGTTAAAAATTCCCTAATTGGCTAATTAGATTGCATAAATGTCAAAAAT
	TAA3-R	ATTTTTGACATTTATGCAATCTAATTAGCCAATTAGGGAATTTTTAACA
pB4	TAA4-F	GGCTATTTAGATTGCATAAATGTCTAAAATTTGAAAAACATACAACGAC
	TAA4-R	GTCGTTGTATGTTTTTCAAATTTTAGACATTTATGCAATCTAAATAGCC
pC1	3-F	AGAAAGAGGGTATGCATATGGATTAAACTGGTGTTATTACAAGCATTAT
	3-R	ATAATGCTTGTAATAACACCAGTTTAATCCATATGCATACCCTCTTTCT
pC2	4-F	ATATGGATTTCACTGGTGTTATTTAAAGCATTATTGATTTAATCAAGAC
	4-R	GTCTTGATTAAATCAATAATGCTTTAAATAACACCAGTGAAATCCATAT
pC3	5-F	GTGTTATTACAAGCATTATTGATTAAATCAAGACTTGCATTCAGGCTTT
	5-R	AAAGCCTGAATGCAAGTCTTGATTTAATCAATAATGCTTGTAATAACAC
pFP	onlyP-F	GCATTTGGATAATTTTTTCAACTAAAAAACAGAGGAAATATTCAACGAC
	onlyP-R	TACTCCTGTAAAATCCATATGCATACCCTCTTTCTATTTAAGAT
	onlyP-F1	TAAAAACATGTATACAAGCATTTGGATAATTTTTTCAACTAAAAAACAG
	onlyP-R1	TTAAATCTATTATTGAAGTAATTACTCCTGTAAAATCCATATGCATACC
	pnlyP-F2	ATTACTTCAATAATAGATTTAATAAAAACATGTATACAAGCA
pNDX1-F	psm-mecRNA-F	ATCTTAAATAGAAAGAGGGTATGCATATGG
	xyl-tet-R	TATTTTAATTATACTCTATCAATGATAGAGTGTCAATATTTTTTTTAG
16S rRNA	16SrRNAF	CTGTGCACATCTTGACGGTA
	16SrRNAR	TCAGCGTCAGTTACAGACCA
psm-mec mRNA	psm-mecF	GGATTTCACTGGTGTTATTAC
	psm-mecR	CCGAAAGCCTGAATGCAAGT
	psm-mecF2	ATCTTAAATAGAAAGAGGGTATGC
	psm-mecR2	GTCGTTGAATATTTCCTCTGTTT
psmα1-2 mRNA	psmaF	TGGGTATCATCGCTGGCATC
	psmaR	TACTTACCAGTGAATTTCTC
psmα3-4 mRNA	psmaF2	ATGGAATTCGTAGCAAAATTATTC
	psmaR2	GCGAAAATGTCGATAATTGCTT
hla mRNA	SA1007-F	GGTGCAAATGTTTCGATTGG
	SA1007-R	CGAAGTCTGGTGAAAACCCTGA
agrA mRNA	SA1844-F	GCCCTCGCAACTGATAATCCT
	SA1844-R	ACCAACTGGGTCATGCTTACG

RNAIII	SAS065-F	GAAGGAGTGTTTCAATGG
	SAS065-R	TAAGAAAATACATAGCACTGAG
sarS mRNA	SA0108-F	CGAGAGAAAATTGCAGAACGTG
	SA0108-R	TGTGATTCACTTTGATCTGCAAGG
fnbA mRNA	fnbA-RT-F	GGGATGGGACAAGACAAAGA
	fnbA-RT-R	GTTGTGCAGTTTGTGGTGCT
hu probes	hu-F	GAAGAATTCTTGGACAAGGTGAATCAATGG
	hu-R	CATGCCATGGGACATTCACCTCCTGAGGTT
psmα promoter	psmA-P-F	GGTGGTACCAGACACTGCATCACGGTACG
	psmA-P-R	TCTTCTAGATTTAATGATGCCAGCGATGA
